# Impact of Prescribed Fire Emissions on Ambient PM_2.5_ and Its Components in the Southeastern US

**DOI:** 10.1021/acsenvironau.6c00072

**Published:** 2026-04-08

**Authors:** Kamal J. Maji, Zongrun Li, Yongtao Hu, Jennifer D. Stowell, Chad W. Milando, Ambarish Vaidyanathan, Gregory A. Wellenius, Patrick L. Kinney, Armistead G. Russell, M. Talat Odman

**Affiliations:** † School of Civil and Environmental Engineering, 1372 Georgia Institute of Technology, Atlanta, Georgia 30332, United States; ‡ Department of Public Health, 4906 Environments, and Society, London School of Hygiene & Tropical Medicine, 15-17 Tavistock Place, WC1H 9SH London, U.K.; § Department of Environmental Health and Center for Climate and Health, 1846 Boston University School of Public Health, 715 Albany Street, Boston, Massachusetts 02118, United States

**Keywords:** prescribed fire, southeastern US, air quality, PM_2.5_, black carbon, organic carbon

## Abstract

Under the framework of the United Nations Sustainable Development Goals, mitigating global PM_2.5_ exposure, which poses a substantial health threat, has become a critical global priority. However, in the southeastern United States (US), the increased use of prescribed fire (PF) or controlled burning as a predominant management tool to prevent destructive wildfires is a major source of PM_2.5_. Despite widespread smoke exposure in this region, the concentrations of PF-induced PM_2.5_ (PF–PM_2.5_), its chemical composition, and its seasonal variability remain poorly understood. Moreover, it is important to quantify the contribution of PF to black carbon in the atmosphere to assess the climatic impact of these burns. We employed a multistage modeling framework to estimate year-round, long-term effects of PF on air quality. The framework integrates a chemical transport model with a data-fusion approach to estimate 24 h average PF–PM_2.5_ and its components [elemental carbon (EC), organic carbon (OC), nitrate (NO_3_^–^), sulfate (SO_4_^2–^), and others] attributable to PF from 2013 to 2021 in 12 southeastern US. We estimated average PF–PM_2.5_ to be 0.50 ± 0.20 μg/m^3^ (mean ± SD), which was approximately 8% of the ambient PM_2.5_. The hot spots with a high PF–PM_2.5_ concentration appeared over southeast Alabama, southwest Georgia, and northwestern Florida. From 2013–2021, average PF–PM_2.5_ contributed 0.85 ± 0.17 μg/m^3^ (12% of ambient PM_2.5_) in Georgia and 0.83 ± 0.16 μg/m^3^ (11% of ambient PM_2.5_) in Alabama. However, during the extensive burning season (January–April), average PF–PM_2.5_ levels accounted for 1.38 ± 0.31 μg/m^3^ (20% of ambient PM_2.5_) in Georgia and 1.11 ± 0.27 μg/m^3^ (16% of ambient PM_2.5_) in Alabama. The 2013–2021 average PF-EC value was 0.067 ± 0.028 μg/m^3^ over the 12 states, which was 27% of ambient EC and increased to 35% of ambient EC during the extensive burning season (0.091 ± 0.045 μg/m^3^). The average PF-OC concentration was also high, 0.23 ± 0.09 μg/m^3^ (13% of ambient OC); however, average concentrations of PF-NO_3_^‑^, PF-SO_4_^2‑^, and other components were very low during the study period. The PF–PM_2.5_ and its components’ concentrations were highly dependent on place and season. This data set can be used as a tool to aid in understanding the interplay between forest management, air quality, and human health in the southeastern US.

## Graphical abstract

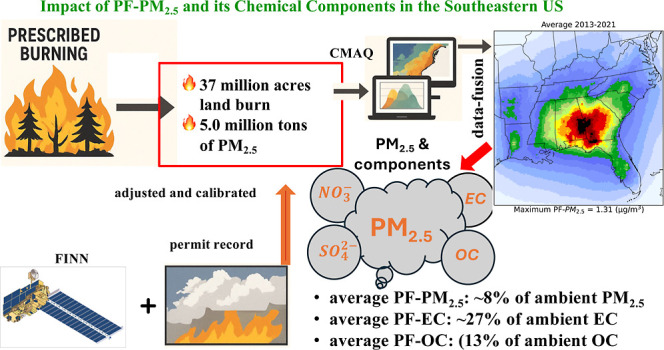

## 1 Introduction

Over the last couple of decades, the primary sources of PM_2.5_ (particulate matter ≤2.5 μm in diameter) emissions have shifted significantly due to factors such as policy interventions, changes in industrial practices, increased social awareness, and advancements in technology.^1^ In the United States (US), a range of policy measures have been implemented during this period to reduce emissions from the electric power, transport, industry, and residential combustion sectors, resulting in a substantial decline in anthropogenic emissions.^2–4^ Despite numerous studies offering new insights into major PM_2.5_ sources,^5,6^ information on the emissions of wildland fire, which includes both wildfire and prescribed fire (PF), remains limited in the US.^7^ Wildland fire, which is strongly influenced by both human activities and climatic conditions, constitutes a major contributor to PM_2.5_ levels in the southeastern US.^8,9^ As wildland fire activity is anticipated to increase in the future,^8^ there is a growing concern of similar increases in adverse health effects associated with smoke exposures.^10^ Additionally, pollutants emitted from wildland fires can stay in the air over long periods of time facilitating their long-range transport impact on populations across continents.^11–13^ PF consisting of smaller and more frequent controlled burns may be used to help reduce the occurrence of large and high-intensity wildfires by reducing excess, extremely dry fuels.^14,15^ Multiple studies have acknowledged the benefits of fuel reduction via PF in mitigating wildfire risk but have also highlighted the dangers of introducing additional smoke into the ambient air.^14,15^ PF generally emits 3–20 times less PM_2.5_ than a wildfire burning in the same area.^16^ For example, in a trade-off analysis of PF against the 2016 Gatlinburg Wildfires, Li et al.^17^ reported that PF consumed 45% less fuel and emitted 52% less PM_2.5_ compared to the wildfire.

PF accounts for a significant portion of biomass burns in the US, averaging 11 million acres per year, 70% of which occurs in the southeastern US.^18^ High PF activity in the southeastern is one of the reasons for the lower wildfire risk compared to the western US.^9^ In the southeastern, the annual prescribed burned area increased by approximately 0.15 million acres from 2000 to 2022.^19,20^ Wildland fire contributes 31% of primary PM_2.5_ emission in the southeastern, in which 81% are coming from PF.^10,21^ PF contributes about 10–15% to the annual average ambient PM_2.5_ levels, with this contribution rising to 20–30% during the peak burning season (January–April).^22,23^ Under a climate change scenario, a ∼5% increase in PF–related PM_2.5_ is projected across the southeastern region from 2015–2019 to 2055–2059, reflecting an increase in the number of burning days across the domain.^24^

PF generally results in lower levels of PM_2.5_ exposure compared to wildfires; however, the frequency of PM_2.5_ exposure from PF is significantly higher.^25^ Although the relative importance of the frequency, duration, and intensity of PM_2.5_ from different types of wildland fires on health is not yet clear,^26^ there is a well-established understanding that long-term exposure to even low concentrations of ambient PM_2.5_ is associated with multiple adverse health outcomes, including mortality.^11,27,28^ Wildland fires emit numerous gaseous and particulate pollutants, including black carbon (BC), organic carbon (OC), carbon monoxide (CO), nitrate, and sulfate into the air.^29–32^ PF contributes 20–40% of organic aerosol (OA) in some US.^33^ Epidemiological and toxicological studies indicate that these compounds may be relatively more hazardous than other PM_2.5_ compounds related to their high potential to inflict oxidative stress and have a major impact on the adverse health effects on humans.^34^ Studies also found that wildfire smoke can be more toxic as compared to emissions from other sources like industries and power generation.^35,36^ Aguilera et al.^36^ found that exposure to wildfire smoke can lead to a 10-fold increase in the risk of respiratory illness-related hospitalizations compared to other sources of PM_2.5_. Chowdhury et al.^6^ found that globally ∼5% of the deaths are associated with chronic exposure to biomass burning-PM_2.5_; this estimate increases to 7.5% when accounting for the higher toxicity of OC and BC from biomass burning smoke. Our recent study demonstrated that PF–PM_2.5_ has positive association with emergency department visits for multiple causes in the southeastern US but at lower rates compared to impacts of wildfire PM_2.5_.^37^

In our previous studies,^23,38^ we quantified PF–PM_2.5_ concentrations at 4 km resolution in Georgia and at 12 km resolution across nine southeastern US. These earlier studies were constrained by approximately 15% missing daily data, did not fully cover all southeastern states, or considered the PM_2.5_-components. In this study, we address these limitations by estimating daily PF–PM_2.5_ and components across 12 southeastern US using the most recent versions of the CMAQ and WRF models at 12 km resolution for the period 2013–2021, with complete daily coverage. In addition, we analyzed the composition of PF–PM_2.5_ for the first time. To evaluate the influence of model resolution of CMAQ on PF–PM_2.5_ estimation, we also conducted a high-resolution (4 km) simulation for Georgia from 2017–2021 and compared results with 12 km resolution modeling. Furthermore, we developed and used a new approach to fuse CMAQ PM_2.5_-components and ambient measured PM_2.5_-components to improve the model performance. With these elements, this study provides a comprehensive assessment of the contribution of PF to PM_2.5_ and its components in the southeastern US and offers important information for the development of integrated strategies for fire management and air quality protection.

## 2 Materials and Methods

We designed a multistage modeling framework to estimate year-round daily PF impacts on air quality in 12 southeastern US (i.e., Alabama, Arkansas, Florida, Georgia, Kentucky, Louisiana, Mississippi, North Carolina, South Carolina, Tennessee, Virginia, and West Virginia) from 2013 to 2021. This framework consists of: (a) derivation of daily PF information from the satellite-derived product Fire INventory from NCAR (FINN); (b) calibration of burned area from FINN with burn area in permit records using a linear regression; (c) simulation of PF contributions to 24 h average PM_2.5_ and its components (BC, OC, NO_3_^–^, and SO_4_^2–^) using the Community Multiscale Air Quality (CMAQ) model; (d) data-fusion of daily observations with CMAQ simulated PM_2.5_ and PM_2.5_ components to reduce modeling uncertainty; and (e) evaluation of the impact of spatial resolution on model performance for PF–PM_2.5_ by running the same models at a 4 km resolution over Georgia from 2017 to 2021 (Figure S1).

### 2.1 Estimation of Prescribed Fire Emissions

Burn permits obtained by private landowners from state agencies often provide a more precise record of burned areas compared to satellite data; however, a complete record of PF in all southeastern states is not available.^9,39–41^ To bridge this data gap, we used burned area information from FINN (version 2.5).^42^ Since FINN captures all types of fire activity,^42^ we implemented a burn-type differentiation algorithm to specifically identify PFs within the FINN data set and applied a linear regression model to adjust and calibrate the FINN-derived prescribed burned area using available burn permit records, enabling more accurate estimation of the prescribed burned area (Figure S1).^43^ Although FINN provides fire emissions, we re-estimated those emissions using the adjusted burned areas in the BlueSky smoke modeling framework.^44^ The reason for this choice is that FINN’s emissions estimates are based on global-average assumptions and lack regional specificity, while BlueSky incorporates US-specific fuel types and emission factors (EFs); therefore, is more likely to produce regionally tailored emissions estimates and better represent regional PF practices. BlueSky uses the Fuel Characteristic Classification System to estimate fuel loads at 1 km resolution. These fuel loads represent the quantity of combustible material (e.g., vegetation) based on fuel characteristics, and the amount of fuel consumed is calculated based on burning efficiency. To estimate fire emissions, BlueSky applies EFs from the Smoke Emissions Repository Application (SERA), which relate the mass of pollutants released to the mass of fuel consumed.^45^ SERA serves as a centralized repository of field- and laboratory-based EFs organized by fuel type for Canada and the U.S., modified combustion efficiency, and burn type.^46^ Thus, emissions depend on various factors, including fire behavior and fuel properties such as the structure and arrangement of fuels, fuel conditions, moisture content, vegetation growth stage, and meteorology.^47^

### 2.2 Air Quality Modeling

Our air quality modeling system consisted of the Weather Research and Forecasting Model (WRF; version 3.9), a numerical weather prediction model, and CMAQ (version 5.4), a chemical transport model (CTM).^48^ CMAQ combines emissions and meteorological parameters and models atmospheric transport, dispersion, chemical transformation, and deposition processes to simulate hourly pollution levels.^49^ Our modeling domain had a horizontal resolution of 12 × 12 km, covering the southeastern US (23.71 to 42.10°N and −95.30 to −73.64°W) with 155 × 150 grid cells. We used the National Emission Inventory (NEI) for all anthropogenic emissions other than PF emissions. To quantify the PF impacts, we generated two sets of concentration fields from two CMAQ simulations between January 2013 and December 2021: a baseline simulation with all emissions (C_all_^sim^) and a second simulation excluding PF emissions (C_no–_ _PF_^sim^). We then calculated the PF contributed pollution (ΔC_PF_^sim^) as

1 $${{\Delta C}_{PF}}^{sim}\left(x,t\right)={C_{\text{all}}}^{sim}\left(x,t\right)-{C_{no-PF}}^{sim}\left(x,t\right)$$

where superscript “sim” indicates simulated concentration and “*x*” and “*t*” denote space and time variability.

The use of multiple grid resolutions in chemical transport modeling reflects a balance between physical realism and computational feasibility. 12 km resolution simulations are widely used for regional air quality assessment because they efficiently capture large-scale emission patterns, synoptic transport, and long-term mean pollutant concentrations.^49,50^ Numerous national exposure and regulatory modeling studies have demonstrated that 12 km resolution is generally sufficient for estimating regional background pollution levels and multiyear average concentrations, particularly where spatial gradients are relatively smooth.^51^ Meanwhile 4 km resolution becomes essential for applications focused on episodic smoke impacts, near-source exposure, and evaluation of short-term air quality exceedances.^52^

In this study, to evaluate the impact of model resolution on CMAQ performance and PF pollution concentration estimates, we applied the model over a domain encompassing the state of Georgia, extending from 28.98°N, −87.82°W to 36.28°N, −79.13°W, using a 180 × 180 grid at 4 km × 4 km horizontal resolution for the period 2017–2021. All other model inputs and parameters were held constant across simulations to isolate the effects of the spatial resolution.

### 2.3 Data Fusion

Modeled concentrations have uncertainties related to emissions inputs, meteorological parameters, and physical/chemical transport processes; therefore, they differ from in situ measurements.^17,53^ To reduce the model biases and error, we followed Friberg et al.^54^ and fused observational data from fixed air quality monitors with daily average PM_2.5_ and its components (BC, OC, NO_3_^–^, and SO_4_^2–^) fields simulated by CMAQ. We removed the monitoring sites data near roads to avoid overestimation during the data-fusion. Over a nine year period, data were collected from 287 PM_2.5_ monitors and 48 speciated PM_2.5_ component monitors across the study area. This resulted in 599 thousand daily PM_2.5_ observations, along with 35, 36, 47, and 47 thousand daily observations of EC, OC, NO_3_^–^, and SO_4_^2–^, respectively. These data were obtained from the EPA’s Air Quality System, the Interagency Monitoring of Protected Visual Environments (IMPROVE), and the Chemical Speciation Network (CSN). The number of PM_2.5_-speciation data points is lower as speciation is limited to IMPROVE and CSN sites some of which operate on a 1-in-3-day sample collection schedule.^55^ For PM_2.5_, data-fusion was conducted between modeled PM_2.5_ (PM_2.5‑__tot_^sim^) and observed PM_2.5_ using a polynomial regression model.^37^ The resultant PM_2.5_ concentration field (PM_2.5‑__tot_^DF^) leverages both temporal accuracy provided by observations and spatial completeness provided by CMAQ simulations. Finally, observation-adjusted PF–PM_2.5_ ( ${{\Delta_{PF}PM}_{2.5-tot}}^{DF}$ ) concentration fields were generated by multiplying the simulated PF concentration ( ${\Delta_{PF}{PM}_{2.5-tot}}^{sim}$ ) with the ratio of fused (PM_2.5‑__tot_^DF^) and simulated (PM_2.5‑__tot_^sim^) concentrations of total PM_2.5_, as follows (Supporting Information, 1.1):

2 $${{\Delta_{PF}PM}_{2.5-tot}}^{DF}\left(x,t\right)={\Delta_{PF}{PM}_{2.5-tot}}^{sim}\left(x,t\right)\times \frac{{{PM}_{2.5-tot}}^{DF}\left(x,t\right)}{{{PM}_{2.5-tot}}^{sim}\left(x,t\right)}$$

For PM_2.5_ chemical speciation (PM_2.5‑spe_), fusion was applied to species-to-total PM_2.5_ ratios rather than to absolute species concentrations. Direct fusion of individual modeled and observed concentrations of species would result in inconsistencies as the sum of fused species would not necessarily equal the fused PM_2.5_ mass. In addition, PM_2.5‑spe_ measurements are typically available every third day, whereas modeled PM_2.5‑spe_ is estimated daily, which could introduce a temporal mismatch if the species were fused independently. To preserve the mass balance, modeled species-to-total PM_2.5_ ratios were adjusted using observed ratios from speciation monitors. This ratio-based approach ensures physically consistent aerosol composition, avoids artificial mass creation or loss, and minimizes bias associated with intermittent speciation sampling. For example, we estimated (PM_2.5‑spe_/PM_2.5‑tot_)^DF^ using (PM_2.5‑spe_/PM_2.5‑tot_)^sim^, the ratio from CMAQ, and (PM_2.5‑spe_/PM_2.5‑tot_)^obs^, the ratio from observation at speciation sites. Then, we multiplied this data-fused ratio field with the data-fused total PM_2.5_ field to get the data-fused PM_2.5‑spe_ field.

3 $${{PM}_{2.5-spe}}^{DF}\left(x,t\right)={{PM}_{2.5-tot}}^{DF}\left(x,t\right)\times {\left[{PM}_{2.5-spe}\left(x,t\right)/{PM}_{2.5-tot}\left(x,t\right)\right]}^{DF}$$

Finally, we calculated the observation-adjusted PF impact for PM_2.5‑spe_ (i.e., OC, EC, etc.) using the following equation:

4 $${{\Delta_{PF}PM}_{2.5-spe}}^{DF}\left(x,t\right)={\Delta_{PF}{PM}_{2.5-spe}}^{sim}\left(x,t\right)\times \frac{{{PM}_{2.5-spe}}^{DF}\left(x,t\right)}{{{PM}_{2.5-spe}}^{sim}\left(x,t\right)}$$

where ${\Delta_{PF}{PM}_{2.5-spe}}^{sim}$ is the simulated PF-associated concentration of PM_2.5_ species and PM_2.5‑__spe_^sim^ is the simulated concentration of PM_2.5_ species from CMAQ.

We aggregated the daily average observation-adjusted PF smoke concentrations across individual states and the 12-state region using a weighted spatial mean approach. This was implemented using the “*exactextractr*” package in R 4.2.3 ^56^ to obtain daily mean values. The annual and seasonal means were then derived by averaging the daily means over the respective periods.

## 3 Results

### 3.1 Prescribed Fire Emissions

A large percentage (∼70%) of wildland fires detected by FINN was classified as PF in the southeastern US. The minimum burn area reported in permits is much smaller than that reported by FINN. For example, in Alabama, the minimum permitted burn area is 0.03 acres, whereas the minimum FINN-detected burned area is 1.18 acres. This discrepancy arises because satellite products have limited ability to detect small fires, resulting in a larger minimum detectable burned area compared with permit records.^57,58^ For the detected fires, a linear regression analysis between the gridded daily burned areas from FINN and permit records from Florida, Georgia, and South Carolina indicated that FINN consistently reports higher daily burned areas, with permit data representing approximately 66% of the FINN estimates (Figure S2). The resulting scaling factor of 0.66 was applied to adjust FINN’s PF burned area estimates for alignment with permit records across all states. Between 2013 and 2021, 37 million acres (∼4.1 million acres/year) of land were treated with PF in the 12 states (Figures 1 and S3), of which about 54.1% was burned during the extensive burning season [January–April (JFMA)], while 22.1% and 23.8% of the area were burned during the least active season [May–September (MJJAS)] and moderate activity season [October–December (OND)], respectively. Of the total prescribed burned area, 84% was on private lands, with the rest on federal lands (Figure S4). Georgia (7.9 million acres), Florida (7.7 million acres), and Alabama (5.8 million acres) are the hot spots of PF among the southeastern states, accounting for approximately 58% of the burned area in the region from 2013 to 2021. The estimated highest prescribed burned area was in 2014 (4.8 million acres) and 2017 (4.6 million acres), whereas the lowest prescribed burned area was in 2020 (3.6 million acres). This coincides with the global COVID-19 pandemic and may be due to lockdowns in effect during JFMA.

**Figure 1:**
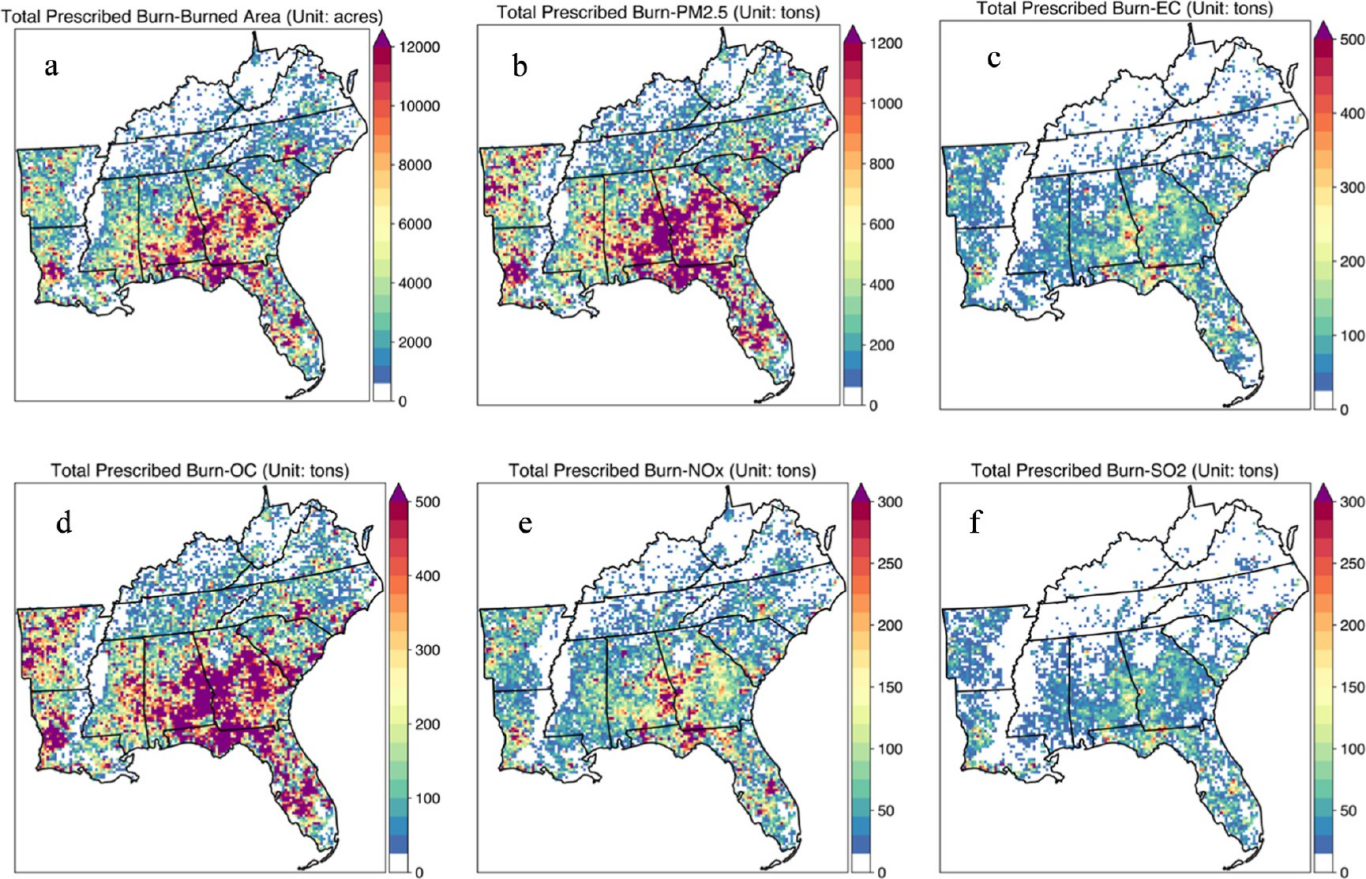
Spatial distribution of (a) adjusted prescribed burned area and corresponding emission of (b) PM_2.5_, (c) elemental carbon (EC), (d) OC, and (e) NO_*x*_ and (f) SO_2_ during 2013–2021 (unit is acres for burned area and tons for PM_2.5_, EC, OC, NO_*x*_, and SO_2_).

During the study period, PF was responsible for emitting 26.4 million tons of CO, 5.3 million tons of volatile organic compounds, 5.0 million tons of PM_2.5_, and 0.60 million tons of nitrogen oxides (NO_*x*_) in the southeastern US. Some of the year-to-year variations in emission rates may be attributed to changes in fuel loads, fuel moisture content, wind speed, and ambient temperature.^59,60^ For instance, in 2021, PF PM_2.5_ emissions were 0.74 tons per acre burned, compared to 0.69 tons per acre in 2015.

### 3.2 Model and Data Fusion Performance

Table S1 summarizes the overall model performance on PM_2.5_ and speciated PM_2.5_ components (i.e., EC, OC, NO_3_^–^, and SO_4_^2–^) using the daily average observations in 12 states during 2013–2021. The CMAQ model generally underestimated mean PM_2.5_ (by ∼20%), EC (by ∼29%), NO_3_^–^ (by ∼10%), and SO_4_^2–^ (by ∼21%). However, CMAQ tended to overestimate the OC (by ∼42%). The CMAQ model may overestimate or underestimate PM_2.5_ species due to uncertainties in input data (e.g., emission inventory), model parametrizations, and inherent limitations. For example, the choice of a particular chemical mechanism may not fully characterize the formation of secondary organic aerosols or meteorological data biases or inaccurate representation of thermodynamic equilibrium models can affect predictions of nitrate, ammonium, and sulfate.^50,61–63^ Data-fusion reduced underestimation of PM_2.5_ (by ∼1.5%), EC (by ∼8%), NO_3_^–^ (by ∼2%), SO_4_^2–^ (by ∼5%), and overestimation of OC (by ∼10%). Data-fused results met the benchmarks of photochemical model performance for PM_2.5_ set by Emery et al.;^64^ *R* ^2^ over the study domain was 0.69 [root mean squared error (RMSE) = 2.43 μg/m^3^; normalized mean error = 20%, normalized mean bias (NMB) = −5.5%] (Figure S5). Data-fusion also improves the accuracy of PM_2.5_ components (lower MNB and MNE). The data-fusion method performance was also evaluated using a comprehensive 10-fold cross-validation analysis. The results (Table S1) indicated that CMAQ with data-fusion performed better compared to CMAQ alone, with larger *R* ^2^ and smaller MB, RMSE, and NMB when compared to observational data.

Finally, to check if the time variation of PF–PM_2.5_ estimates agrees with observations, observed PM_2.5_ is plotted versus estimated PF–PM_2.5_ (Figure S6). Note that most of the data points are concentrated around a PM_2.5_ of ∼10 μg/m^3^ and very small PF–PM_2.5_, suggesting a background PM_2.5_ level of ∼10 μg/m^3^ without the PF impact. On the other hand, PF impacts manifested as larger PF–PM_2.5_ values also show up as increases in PM_2.5_ observations; this builds confidence in the PF–PM_2.5_ estimates. Some CMAQ estimates of PF–PM_2.5_ exceed observed PM_2.5_ (points below the diagonal in the left panel of Figure S6), which is unrealistic. Data fusion corrects most of those cases (right panel of Figure S6). The remaining points below the diagonal are cases in which smoke is predicted to hit the monitor but misses it. The data points along the *y*-axis above the background are cases where observed high PM_2.5_ is due to a source other than PF. Some of them may also be cases when smoke is predicted to miss the monitor but hits it. Note that data fusion increases the overall correlation between observed PM_2.5_ and PF–PM_2.5_ and reduces RMSE.

### 3.3 Prescribed Fire-PM_2.5_

There are high spatial and temporal variations in the annual average PF–PM_2.5_ concentrations across the southeastern US, covering the period from 2013 to 2021 (Figure 2). Over the nine year period across the 12 states, average PF–PM_2.5_ was 0.50 ± 0.20 (mean ± SD) (median: 0.46) μg/m^3^ (we follow this reporting structure below), which was 8% of the ambient PM_2.5_. The highest annual average PF–PM_2.5_ was 0.63 ± 0.25 (0.59) μg/m^3^ seen in 2017, followed by 0.57 ± 0.23 (0.51) μg/m^3^ in 2021, which were around 10% and 9% of ambient PM_2.5_. The hot spots with high PF–PM_2.5_ pollution were over southeast Alabama, southwest Georgia, and northwestern Florida. From 2013 to 2021, average PF–PM_2.5_ contributed 0.85 ± 0.17 (0.86) μg/m^3^ (12%) to ambient PM_2.5_ in Georgia and 0.83 ± 0.16 (0.84) μg/m^3^ (11%) in Alabama (Figure 2 and Table S2); however, in 2017, contributions to ambient PM_2.5_ reached 1.13 ± 0.24 (1.18) μg/m^3^ (16%f) and 1.01 ± 0.23 (0.98) μg/m^3^ (14%) in Georgia and Alabama, respectively.

**Figure 2:**
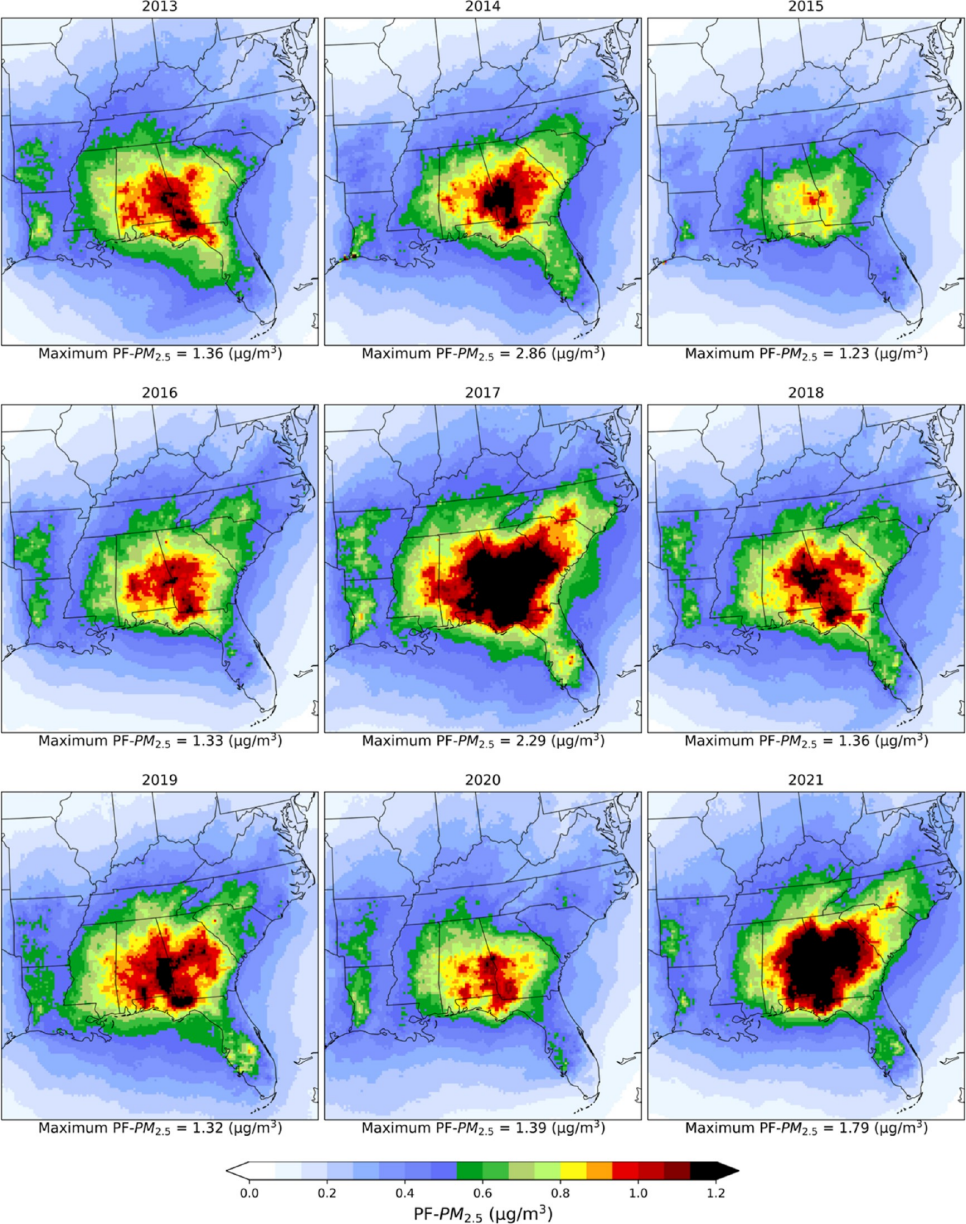
Spatial distributions of yearly average prescribed fire PM_2.5_ concentrations (μg/m^3^) during 2013–2021.

Seasonal variability was seen in the spatial distribution of PF–PM_2.5_, as the area treated with PF depends on seasonal weather conditions and local regulations (Figure 4). Across the 12 states, during JFMA 2013–2021 average PF–PM_2.5_ was 0.70 ± 0.32 (0.61) μg/m^3^ (11% of ambient PM_2.5_). Highest JFMA average PF–PM_2.5_ was 0.91 ± 0.50 (0.78) μg/m^3^ (15% of ambient PM_2.5_) in 2017. Alabama, Georgia, and South Carolina experienced higher PF–PM_2.5_ levels than other states during JFMA 2013–2021:1.11 ± 0.27 μg/m^3^ (16% of ambient PM_2.5_) in Alabama, 1.38 ± 0.31 μg/m^3^ (20% of ambient PM_2.5_) in Georgia, and 0.99 ± 0.14 μg/m^3^ (15% of ambient PM_2.5_) in South Carolina (Table S3). In JFMA 2017, the contribution of PF–PM_2.5_ reached approximately 25% of ambient PM_2.5_ in these three states. On the days with the highest burned area, PF–PM_2.5_ contributed up to 75% to ambient PM_2.5_. For example, on March 8, when the highest burned area in 2021 was reported (125,500 acres over the 12 states), PF–PM_2.5_ contributed ∼9.0 μg/m^3^ in Georgia, Alabama, and South Carolina, comprising approximately 75% of the ambient PM_2.5_ (Figure S7).

**Figure 3:**
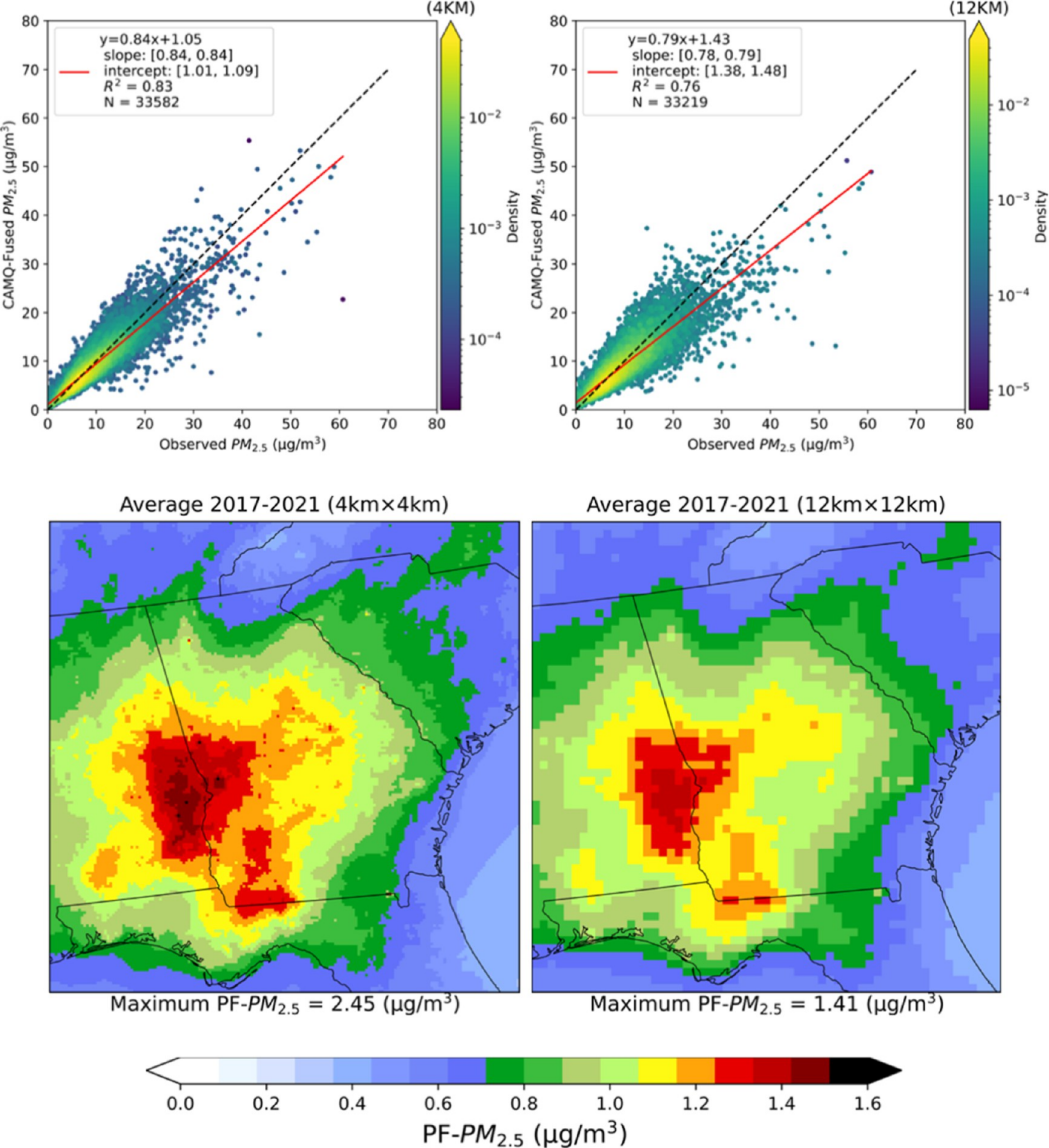
Density scatterplots of daily observed vs data-fused total PM_2.5_ at 4 km × 4 km (top left) and 12 km × 12 km (top right) CMAQ model resolutions in Georgia, and prescribed fire PM_2.5_ at 4 km × 4 km (bottom left) and 12 km × 12 km (bottom right) resolutions in Georgia and surrounding areas.

**Figure 4:**
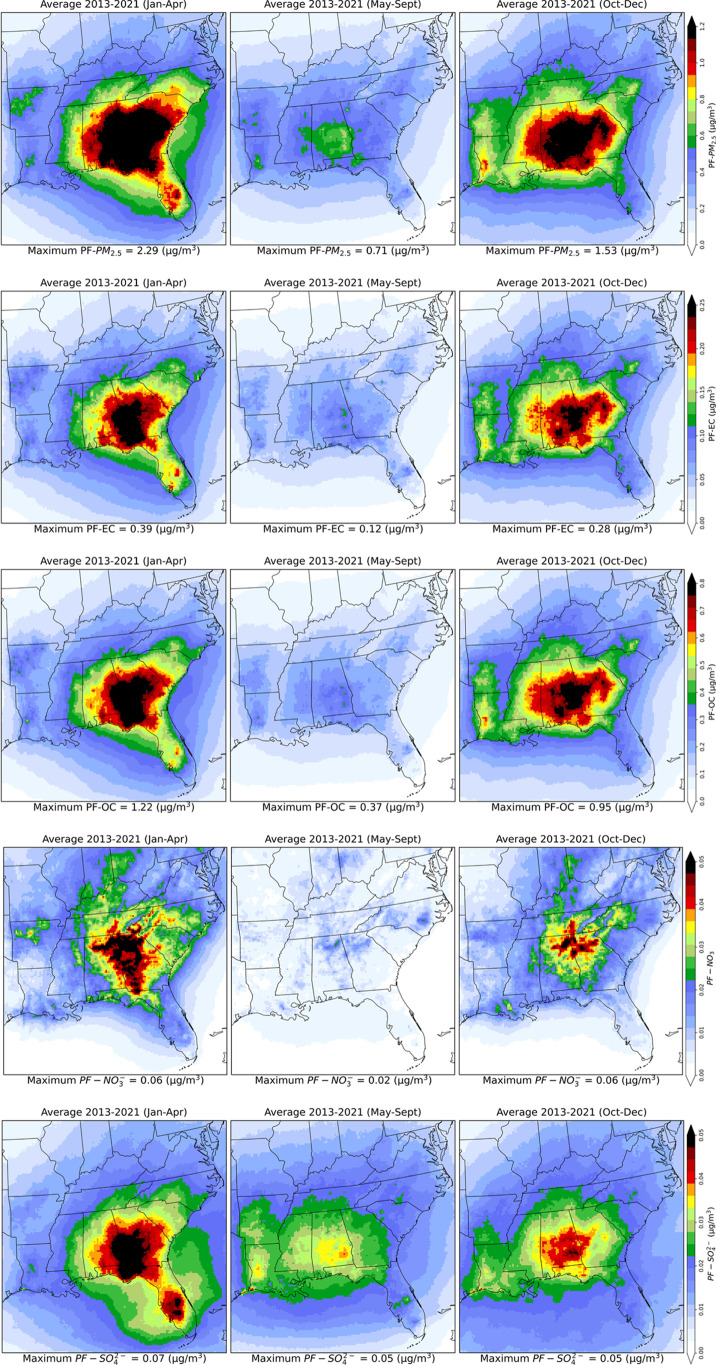
Spatial distributions of seasonal average prescribed fire PM_2.5_ (1st row), EC (2nd row), OC (3rd row), NO_3_^–^ (4th row), and SO_4_^2–^ (bottom row) concentration (mg/m^3^) during 2013–2021. January–April (left column) is the extensive burning season, May–September (center column) is the low burning season, and October–December (right column) is the moderate burning season.

During MJJAS, some states imposed a ban on prescribed burning in certain countries. This is due to the increased likelihood of elevated O_3_ levels, combined with the presence of dry fuels and high wind speeds, which can make prescribed fire difficult to control.^38,65,66^ The nine year 12-state MJJAS average PF–PM_2.5_ was 0.36 ± 0.10 (0.36) μg/m^3^, which accounted for 5% of the ambient PM_2.5_. During MJJAS, Alabama experienced higher exposure to PF–PM_2.5_, averaging 0.54 ± 0.07 μg/m^3^, compared to Georgia. This is likely because Alabama has fewer restrictions on prescribed burning during the summer. Georgia PF–PM_2.5_ was on average 0.45 ± 0.07 μg/m^3^ during MJJAS despite burning restrictions, potentially resulting from the transport of smoke from surrounding states (Table S4).

During OND, average PF–PM_2.5_ was 0.64 ± 0.23 (0.6) μg/m^3^ over the region during 2013–2021. PF–PM_2.5_ values in Alabama, Georgia, and Mississippi were relatively higher than those in other states during this season. In these three states, average PF–PM_2.5_ was 1.13 ± 0.18 μg/m^3^, 0.97 ± 0.2 μg/m^3^, and 0.82 ± 0.14 μg/m^3^, respectively, during OND 2013–2021 (Table S5). The average PF–PM_2.5_ levels during OND in Arkansas, Kentucky, and Tennessee were higher than the JFMA average due to a higher number of prescribed burns during OND, facilitated by favorable weather conditions in those states.^67,68^ In 2020, PF–PM_2.5_ levels in the study region were higher during OND than JFMA. This shift might be linked to COVID-19, as most prescribed burns were rescheduled from January–April to October–December.^69^

The maximum grid-cell daily average PF–PM_2.5_ was 53 μg/m^3^, higher than the EPA daily air quality standard of 35 μg/m^3^; moreover, PF is responsible for ∼1.6% of grid cell-days with a daily contribution of ≥10% of daily national ambient PM_2.5_ standard (≥3.5 μg/m^3^) during 2013–2021. In 2017, more than 2.6% of all grid cell days exceeded PF–PM_2.5_ of 3.5 μg/m^3^ (Figure S8). During the study period, a substantial number of days with daily average PF–PM_2.5_ concentrations of ≥3.5 μg/m^3^ were observed across several states: 175 days in Alabama, 170 days in Georgia, 112 days in South Carolina, 78 days in Tennessee, and 66 days in North Carolina.

We also ran CMAQv5.4 at 4 km resolution over Georgia (all other parameters being the same) from 2017 to 2021 to further investigate the uncertainty due to grid resolution. We have seen the 4 km resolution model performance in Georgia (*R* ^2^: 0.83; RMSE: 1.97 μg/m^3^; NMB: −4%; MNB: 0.4%; MNE: 17%) improve over the 12 km resolution model (*R* ^2^: 0.76; RMSE: 2.35 μg/m^3^; NMB: −5.3%; MNB: 1%; MNE: 21%). Five year average PF–PM_2.5_ in Georgia was 1.06 ± 0.23 (1.09) μg/m^3^ with 4 km resolution, ∼10% higher than PF–PM_2.5_ with 12 km resolution [0.96 ± 0.18 (0.96) μg/m^3^] (Figure 3).

### 3.4 Prescribed Fire EC

The PF-induced EC (PF-EC) concentrations change over space and time across the southeastern US, and the relative contribution of PF-EC to the total EC also changes significantly during 2013–2021 (Figure 5). The simulated nine year-averaged PF-EC values were 0.067 ± 0.028 (0.061 μg/m^3^) over the 12 states, which was 27% of ambient EC. The highest annual average PF-EC, 0.089 ± 0.039 (0.083) μg/m^3^, was seen in 2021, followed by 0.085 ± 0.036 (0.079) μg/m^3^ in 2019, which were around 31% and 28% of ambient EC, respectively. Noticeably larger values of PF-EC (>0.12 μg/m^3^) were estimated in Georgia and Alabama, around 37% of ambient EC; however, in 2019, average PF contribution to EC reached 0.16 μg/m^3^ (43% of ambient EC) (Table S6).

**Figure 5:**
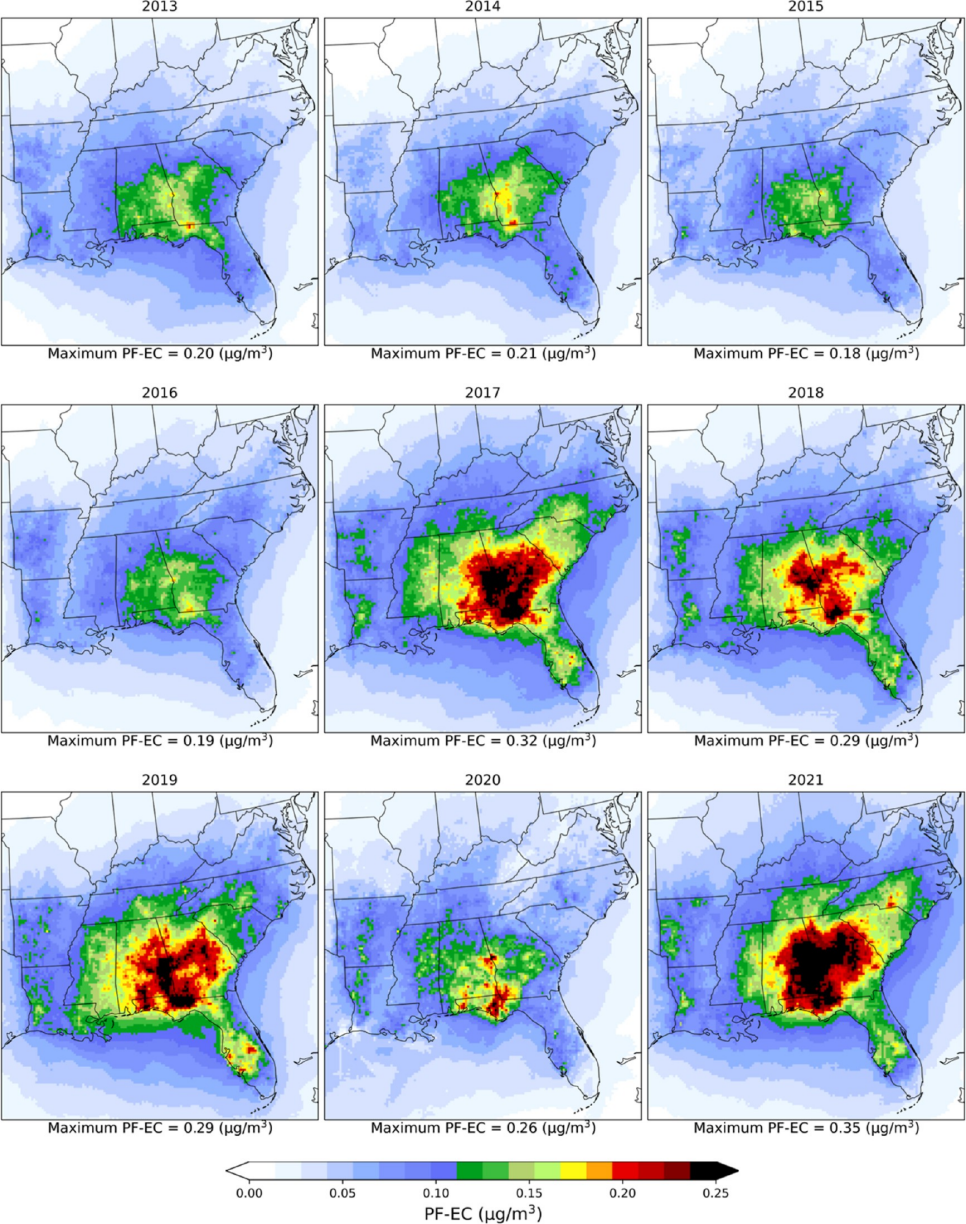
Spatial distributions of yearly average prescribed fire EC concentrations (μg/m^3^) during 2013–2021.

High seasonal variability was seen in the spatial distribution of PF-EC (Figure 4). Across the 12 states, JFMA-average PF-EC was 0.091 ± 0.045 (0.075) μg/m^3^ (35% of ambient EC) during 2013–2021, with the highest average PF-EC of 0.112 ± 0.060 (0.088) μg/m^3^ (40% of ambient EC) seen in 2021. Alabama, Florida, and Georgia experienced high PF-EC levels during the extensive burning season: 0.144 ± 0.041 μg/m^3^ (43% of ambient EC) in Alabama, 0.131 ± 0.051 μg/m^3^ (46% of ambient EC) in Florida, and 0.184 ± 0.045 μg/m^3^ (47% of ambient EC) in Georgia (Table S7). The nine year 12-state MJJAS average PF-EC was 0.042 ± 0.013 (0.041) μg/m^3^, which accounted for 20% of the ambient EC. During MJJAS, Alabama, Arkansas, and Mississippi experienced higher PF-EC (∼27% of ambient EC) because these states have fewer restrictions on prescribed burning during the summer. During OND, prescribed burning in Alabama, Georgia, and Mississippi was relatively higher than in other states. In these three states, average PF-EC was 0.182 ± 0.039 μg/m^3^, 0.154 ± 0.041 μg/m^3^, and 0.127 ± 0.027 μg/m^3^, respectively, during OND 2013–2021, which was around 40% of ambient EC (Table S8). The maximum grid-cell PF-EC was 19 μg/m^3^; however, only ∼0.44% of grid cell-days had ≥1 μg/m^3^ of PF-EC over the study period. In 2021, PF-EC exceeded 1 μg/m^3^ in ∼0.84% of all grid cells.

### 3.5 Prescribed Fire OC

During 2013–2021, the average PF-induced OC (PF-OC) concentration was 0.23 ± 0.09 (0.21) μg/m^3^ over the 12 states, which was 13% of ambient OC. Yearly mean concentration was dependent on the prescribed burned area and associated meteorological conditions (Figure 6). Higher annual average PF-OC was seen in 2017 [0.27 ± 0.11 (0.25) μg/m^3^] and 2019 [0.30 ± 0.13 (0.27) μg/m^3^], which accounted for ∼15% of ambient OC. The 2013–2021 average state level PF-OC was relatively high in Alabama (0.39 ± 0.08 μg/m^3^), Florida (0.25 ± 0.11 μg/m^3^), and Georgia (0.40 ± 0.08 μg/m^3^), accounting for ∼18% of ambient OC, however the annual average PF-OC reached ∼23% of ambient OC in 2019 in those states (Table S10).

**Figure 6:**
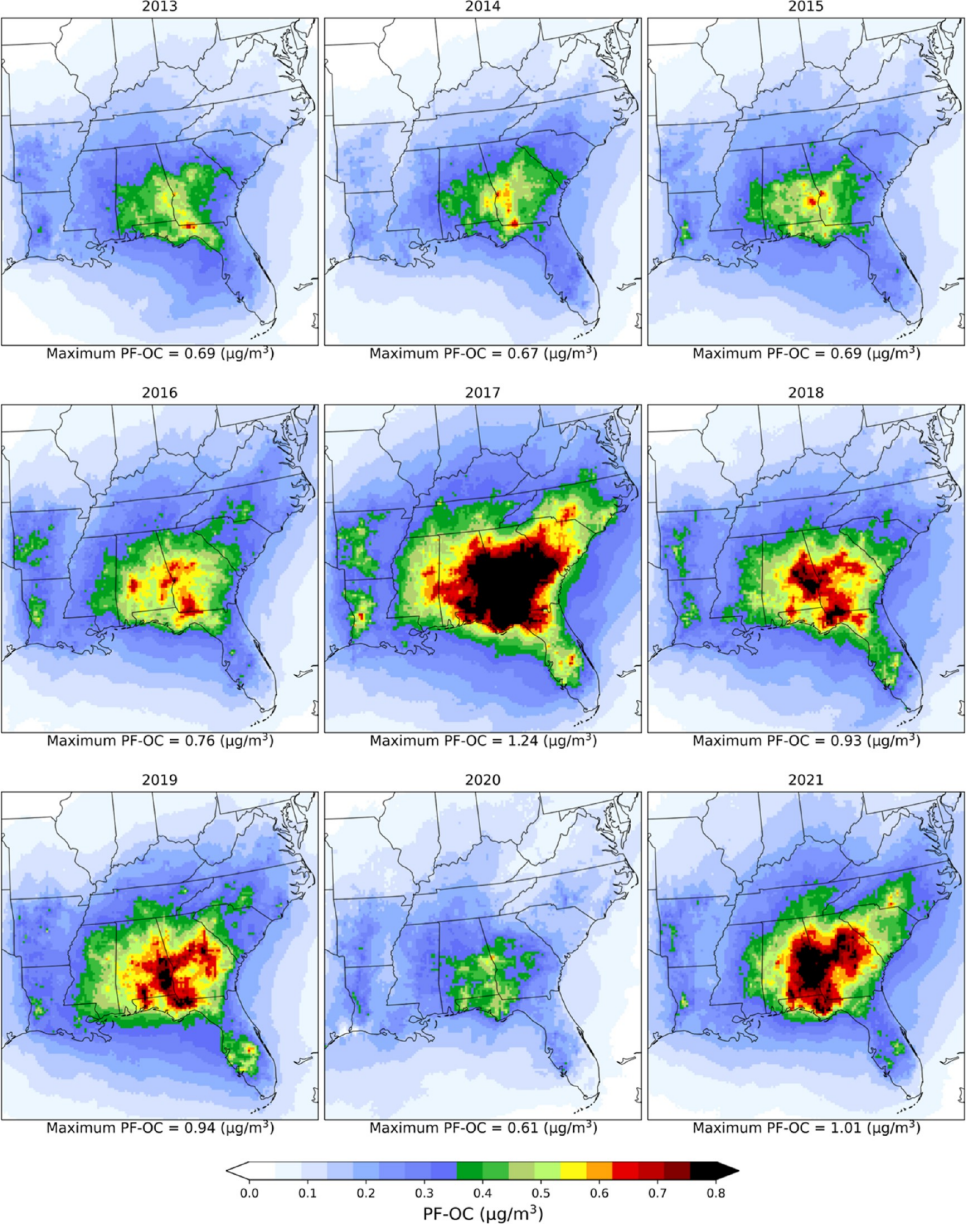
Spatial distributions of yearly average prescribed fire OC concentrations (μg/m^3^) during 2013–2021.

During JFMA, over the 12 states, average PF-OC was 0.31 ± 0.15 (0.26) μg/m^3^, which accounted for 21% of the ambient OC. During this season, Alabama (0.48 ± 0.13 μg/m^3^), Florida (0.42 ± 0.16 μg/m^3^), Georgia (0.62 ± 0.14 μg/m^3^), and South Carolina (0.44 ± 0.07 μg/m^3^) experienced higher levels of PF-OC than other states, reaching ∼28% of the ambient OC (Figure 4 and Table S11). During MJJAS, average PF-OC contributed only 7% to the ambient OC; however, during OND, average PF-OC contribution increased again, reaching 19% (0.33 ± 0.13 μg/m^3^) of the ambient OC (Table S13). As prescribed burning shifted from JFMA to OCD in 2020 due to COVID-19, higher levels of PF-OC were seen during OND, especially in Alabama (0.81 ± 0.17 μg/m^3^), Georgia (0.63 ± 0.21 μg/m^3^), and Mississippi (0.62 ± 0.10 μg/m^3^), which accounted for ∼30% of ambient OC. The maximum grid-cell daily average PF-OC was 75 μg/m^3^; however, only ∼3.6% of grid cell-days had ≥1 μg/m^3^ of PF-OC over the study period.

### 3.6 Prescribed Fire NO_3_^–^ and SO_4_^2–^

As shown above, on average, approximately 13% of PF–PM_2.5_ consisted of PF-EC and 46% of PF-OC. There is roughly another 37% of organic mass associated with PF-OC. The remainder consists of sulfate, nitrate, ammonium, crustal material, soils, and metals. Here we will focus on the sulfates and nitrates due to their impact on human health and the climate.^70^ Concentrations of PF-NO_3_^‑^ and PF-SO_4_^2‑^ were very low throughout the domain (Figure 7). During the study period, average PF-NO_3_^‑^ was 0.009 ± 0.003 μg/m^3^ over the 12 states, which was only 3% of ambient NO_3_^–^; however, in Alabama and Georgia, the contribution was around 5%. During JFMA, PF-NO_3_^‑^ concentration was 0.017 ± 0.006 μg/m^3^, almost twice the annual average, due to most of the burns being conducted in this season (Figure 4). Similarly, low concentration of PF-SO_4_^2‑^ was seen during the study period, ∼1.5% of ambient SO_4_^2–^ (0.02 ± 0.01 μg/m^3^) over the 12 states. In Alabama and Georgia, PF-SO_4_^2‑^ contributed approximately 3% to ambient SO_4_^2–^ concentrations. In Alabama, Florida, and Georgia, PF-SO_4_^2‑^ levels reached 0.03 μg/m^3^ during JFMA (Tables S14–S21).

**Figure 7:**
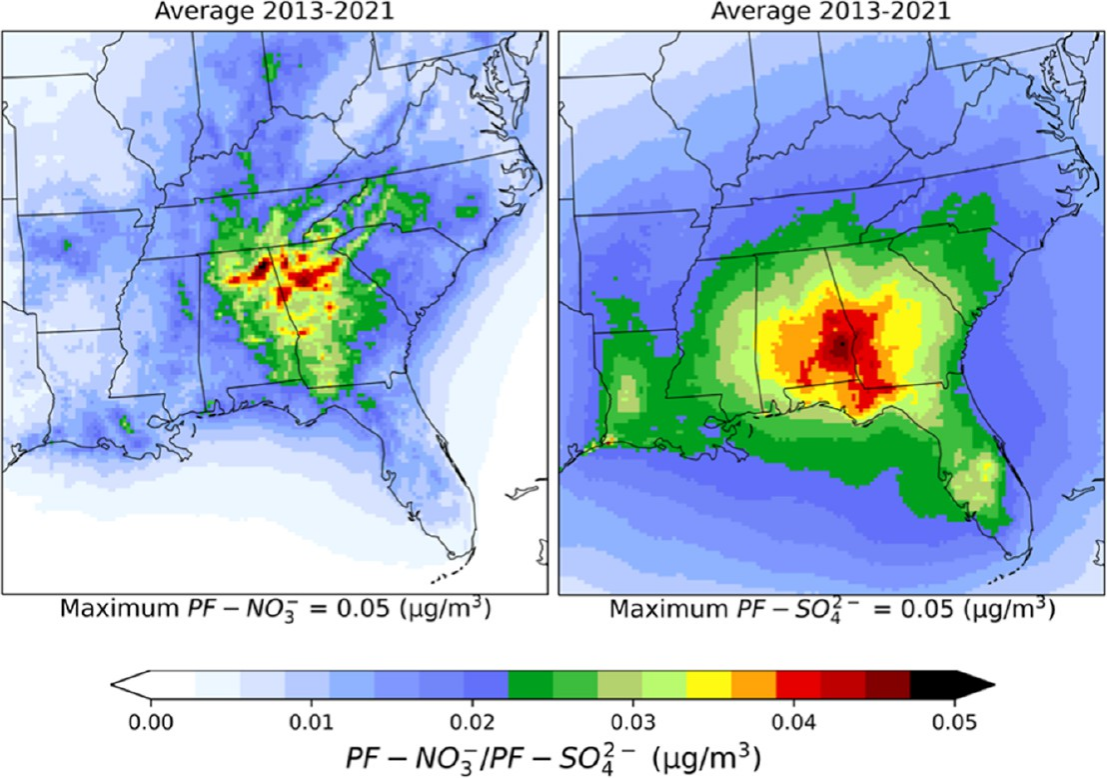
Spatial distributions of nine year average (from 2013–2021) prescribed fire nitrate (NO_3_^–^) and sulfate (SO_4_^2–^) concentrations (μg/m^3^).

## 4 Discussion

This study integrated satellite observed and state-reported PF data and used them in the BlueSky modeling framework to predict PF emissions in the southeastern US during 2013–2021. A CTM at 12 km resolution and a data-fusion method was employed to estimate the impact of PF to air quality. The performance of the CTM and data-fusion was evaluated through a 10-fold cross-validation approach and was found to be acceptable according to commonly used criteria. Further, the temporal variation of estimated PF–PM_2.5_ agreed with that of observed PM_2.5_. We found that a significant portion (≥10%) of the total PM_2.5_ mass and its key chemical constituents (EC and OC) originated from PF in certain southeastern states and during specific years and seasons. At present, there is a broad and coordinated effort among federal land management agencies to expand the use of PF as a strategy to reduce fuel loads and mitigate the risk of large, catastrophic wildfires. However, the anticipated increase in PF will inevitably lead to additional smoke exposures. This will introduce added complexity to air quality management by altering the spatial and temporal patterns of smoke levels and extending the duration of smoke exposures experienced by some communities.^71^ In this context, our findings are important because not only increased total PM_2.5_ in the air but high proportions of EC and OC in PM_2.5_ can lead to increased risk of all-cause mortality among older adults.^34^

PF is widely recognized as one of the most effective strategies to reduce wildfire risk and sustain biodiversity in US.^17,72–74^ Studies have found lower concentrations of smoke-derived carbonaceous PM_2.5_ in the southeastern US during megafire years (e.g., 2017 and 2018) compared with the Western US.^75^ However, this does not necessarily imply that with more extensive use of PF, the US improved air quality in the southeastern. PF generates more consistent smoke pollution with lower but still elevated carbonaceous PM_2.5_ levels over time. Jin et al. estimated 4702 smoke attributable deaths per year in the southeastern US, exceeding the combined totals in the western US (1568 deaths/year) and northeastern US (1192 deaths/year).^75^ In our estimate, PF–PM_2.5_ is responsible for 2552 premature deaths/year in the Southeast.^38^ Several factors contribute to this outcome, including the frequent, lower-intensity burns in the Southeast that produce regular smoke pollution, unlike the sporadic but intense wildfires of the western US. Moreover, PF smoke in the Southeast often impacts densely populated areas such as Atlanta and Charlotte, whereas Western wildfires typically affect more remote forested regions, despite their smoke occasionally reaching urban centers like Los Angeles or San Francisco.^40,76,77^

The multistage modeling framework used here can reduce the overall uncertainty in the PF smoke concentration estimation; however, the study is still subject to some limitations.

First, reliance upon FINN burned area is a significant drawback as FINN cannot capture small burns, and factors such as cloud cover and the timing mismatch between prescribed burn periods and satellite overpasses further reduce the probability of detection.^78^ Nowell et al.^78^ reported that satellite-derived burned area products can misrepresent the spatiotemporal variability of PFs and tend to underestimate the total burned area compared with permit records in Florida. They reported an annual average prescribed burned area of 1.36 million acres during 2004–2015 based on Florida Forest Service records, whereas our estimate was 0.85 million acres per year during 2013–2021. However, FINN overestimated the burned area for the detected burns by 9% in Florida during 2013–2021.^43^

Second, the PF emissions calculated by BlueSky with the SERA data set were used, which may be quite different from other emission inventories. The SERA data set extensively combines EFs (g kg^–1^) of select pollutants (CO, CO_2_, CH_4_, NOx, total PM, PM_2.5_, and SO_2_) and is influenced by combustion phase, burn type, and fuel type. Of the 12, 533 records in the database, over a third (*n* = 5637) are associated with 23 air pollutants. Direct PF–PM_2.5_ emissions from FINN, which we did not use, were 5% lower than our estimates,^43^ while PF–PM_2.5_ emissions in the National Emissions Inventory (NEI) were 45% lower. These differences varied from state to state and year to year; for example, in Alabama, our PF–PM_2.5_ emissions estimate for 2017 was 92 thousand tons, whereas FINN and NEI emissions were 113 (higher) and 37 thousand tons (much lower), respectively. We have not found any direct comparison of current biomass burning emission inventories for PF, but we expect them to have large uncertainties based on what has been reported for wildfires.^79–82^ For example, Li et al.^83^ reported that the total mass of PM_2.5_ emissions from the 2020 August Complex wildfire in California, was underestimated by the Global Fire Assimilation System (GFA) (164 Gg) and Global Fire Emissions Database (GFED) (455 Gg) compared to FINN (703 Gg), whereas Global Fire Emissions Database (QFED) (1171 Gg) and the Blended Global Biomass Burning Emissions Product (GBBEPx) (1211 Gg) overestimated. Similar observations for wildfires were reported by other studies.^42,84,85^ The discrepancies between different emission inventories depend on location, season, and fuel consumption^86^ and there is no “gold standard” emission inventory for biomass burning.

Third, the limitation arises from fuel consumption estimates in BlueSky, which uses the CONSUME model. For 1 h and 10 h fuels, CONSUME assumes complete consumption during a burn, independent of meteorological or site-specific conditions, based on field observations. Consumption of 100 h fuels is estimated using empirical relationships parametrized by terrain slope, wind speed, and fuel moisture.^87^ In addition, BlueSky relies on a default spatial sampling approach to infer the fuel type and fuel load, which may not fully represent heterogeneity across the burned area. This simplification introduces additional uncertainty, as more accurate fuel characterization would require detailed fire perimeter information, which is not currently available in the FINN product.

The model resolution of CTMs and their configuration play a crucial role in the uncertainty associated with the estimation of PF smoke concentrations. For example, in 2017, Maji et al.^23^ estimated average PF–PM_2.5_ to be 1.39 μg/m^3^ in Georgia using 4 km resolution, but only 1.23 μg/m^3^ using 12 km resolution with CMAQv5.3.^38^ This study found a 2017 average PF–PM_2.5_ of 1.13 μg/m^3^ using a 12 km resolution with CMAQv5.4. This highlights the potential impact of grid resolution and model version on estimated concentrations and underscores the importance of consistent configuration in comparative analyses. With a 4 km model resolution, Huang et al.^88^ reported an average PF–PM_2.5_ concentration of 1.06 μg/m^3^ in Georgia during JFMA 2017, based on emissions calculated using only permit-recorded burn area data. In contrast, Maji et al.^38^ reported a much higher PF–PM_2.5_ concentration of 2.40 μg/m^3^ in Georgia during JFMA 2017, derived from emissions calculated using adjusted FINN-burned area data. The lower value reported by Huang et al.^88^ is likely attributable to the exclusion of unrecorded fires.

In our study, the improved model performance and higher PF–PM_2.5_ results of the 4 km simulation relative to the 12 km configuration arise from a combination of interacting physical, numerical, and representational factors. First, at 4 km resolution, emissions are injected into much smaller grid cells, reducing artificial dilution and allowing higher, more realistic near-source concentrations to be simulated. A 4 km resolution better captures local-scale meteorological phenomena, such as localized wind patterns, sea breezes, or urban heat islands, which significantly affect pollutant dispersion, whereas at 12 km resolution localized variations in transport and mixing may be missing.^89–92^ Second, air quality models simulate highly nonlinear chemical reactions between pollutants. At 4 km resolution, high concentrations of precursors in localized areas may lead to faster reaction and more complete formation of secondary PM_2.5_, whereas at 12 km resolution, averaged precursor concentrations over larger areas potentially lead to slower reaction and underestimate the formation of secondary pollutant formation.^93^ Third, land-use and land-cover heterogeneities strongly influence dry deposition, turbulence, surface roughness, and boundary-layer development. Coarse grids smooth over urban–rural contrasts, vegetation variability, and soil moisture gradients that affect plume dispersion and deposition. At 4 km resolution, CMAQ more realistically represents these surface heterogeneities, improving simulation of near-surface concentrations and diurnal variability.^94,95^ Fourth, the CMAQ employs Eulerian advection schemes that inherently introduce numerical diffusion. As grid-cell size increases, this diffusion becomes more pronounced, artificially smoothing pollutant gradients and dispersing plume mass too rapidly. At finer resolution, numerical diffusion is substantially reduced, allowing sharper plume edges and higher concentration maxima to be preserved during transport.^96,97^ Finally, surface monitors sample air quality at spatial scales on the order of hundreds of meters to a few kilometers. When compared with 12 km grid averages, substantial representativeness error is introduced, particularly for spatially heterogeneous smoke plumes. The 4 km grid more closely aligns with the spatial representativeness of monitoring sites, reducing mismatch between simulated and observed concentrations and improving correlation, RMSE, and bias statistics.^98,99^

Despite some uncertainty, this study gives insightful quantitative information on the contribution of PF to PM_2.5_ and its components. This information would be useful in future epidemiological studies for a better understanding of PF impacts on public health in the southeastern US. Furthermore, by rationalizing unplanned wildfires as “natural”, the federal government excludes pollutants from such events in air quality compliance calculations, while encouraging states to strictly regulate pollutants from “anthropogenic” PFs. Therefore, a trade-off study comparing the impacts of PFs and wildfires on air quality and health across the southeastern United States is critical for optimizing prescribed burn area management.

## Supplementary Material



## Data Availability

The source data for the study are deposited in Zenodo. All the data are freely available on https://zenodo.org/records/13380570. No custom code was used in this analysis. All code used is available upon request. Requests for materials should be addressed to Dr. M. Talat Odman.

## Notes

Prescribed burn detection method; data-fusion method, burn area, and corresponding emission; seasonal burn area and impacts; density scatterplots of observed vs model; maximum concentration and smoke days; model performances; and seasonal data (PDF)
