# Supplementary material for: Impact of Prescribed Fire Emissions on Ambient PM2.5 and Its Components in the Southeastern US
Source: ACS Environ Au. 2026 Apr 8;6(3):523–38. doi: 10.1021/acsenvironau.6c00072 (PMC13195466; doi:10.1021/acsenvironau.6c00072)
Supplement: Supplementary file 1 [file vg6c00072_si_001.pdf]

Supporting Information

Impact of Prescribed Fire Emissions on Ambient $PM_{2.5}$ and its Components in the Southeastern US

Kamal J. Maji[a,b], Zongrun Li[a], Yongtao Hu[a], Jennifer D. Stowell[c], Chad W. Milando[c], Ambarish Vaidyanathan[a], Gregory A. Wellenius[c], Patrick L. Kinney[c], Armistead G. Russell[a], and M. Talat Odman[a,*]

[a] *School of Civil and Environmental Engineering, Georgia Institute of Technology, Atlanta, Georgia 30332, USA*

[b] *Now at: Department of Public Health, Environments, and Society, London School of Hygiene & Tropical Medicine, 15-17 Tavistock Place, WC1H 9SH, London, UK*

[c] *Department of Environmental Health and Center for Climate and Health, Boston University School of Public Health, 715 Albany Street, Boston, MA 02118, USA*

[*] *Corresponding author: M. Talat Odman, School of Civil and Environmental Engineering, Georgia Institute of Technology, Atlanta, Georgia 30332, USA, Email: talat.odman@ce.gatech.edu*

Number of pages: 33

Number of figures: 8

Number of tables: 21

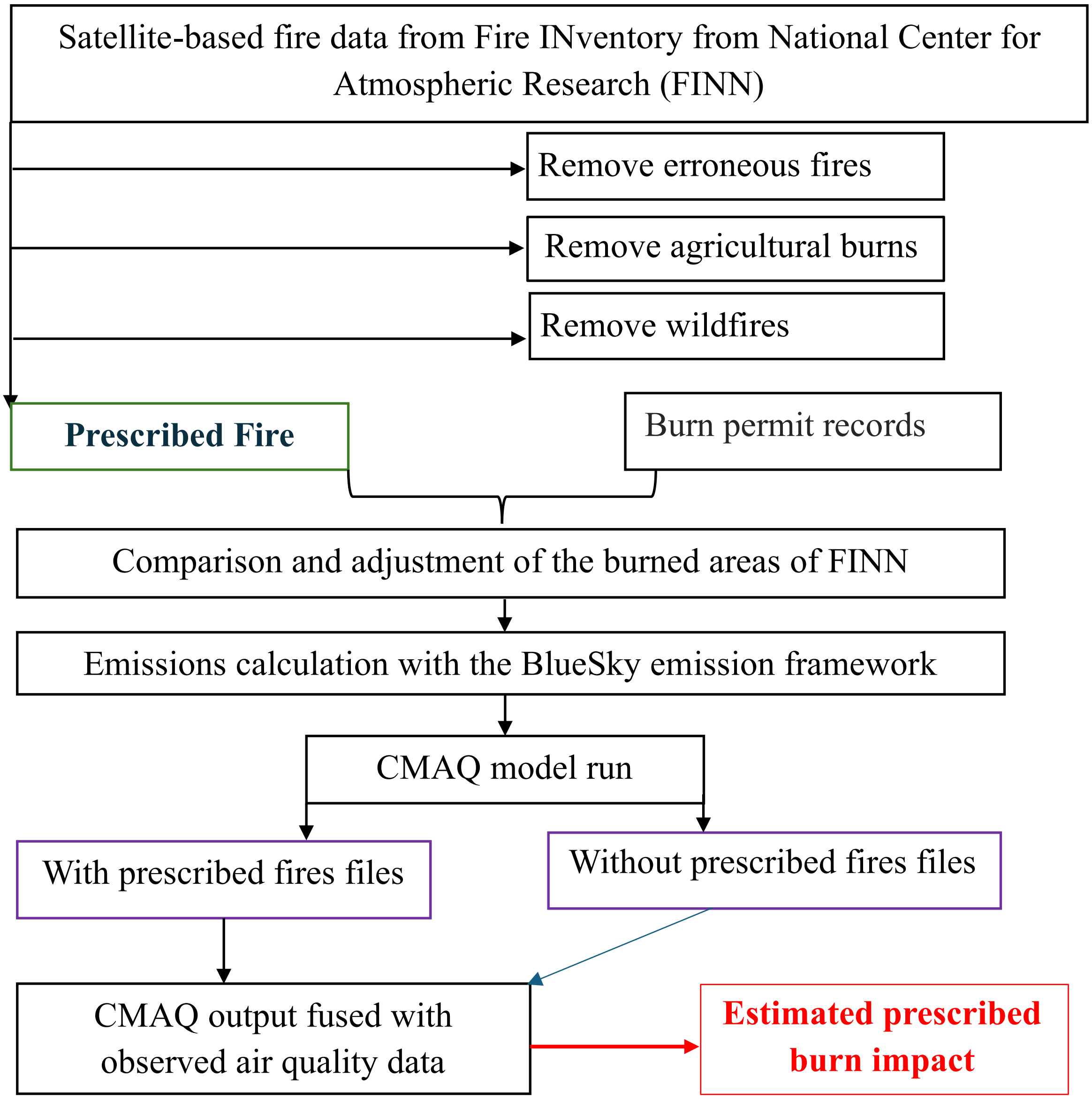

Figure S1. A simplified flow chart of the prescribed burn-associated air quality assessment method.

1.1 Data Fusion Method:

Conceptual motivation: Chemical transport models (CTMs) provide continuous spatiotemporal fields that are physically consistent with emissions, transport, chemistry, and deposition processes. However, CTM outputs often exhibit systematic biases due to uncertainties in emissions, meteorology, and chemical mechanisms (Appel et al., 2021; Gao and Zhou, 2024). Conversely, surface monitoring data provide accurate point measurements but are spatially sparse and cannot independently generate gridded concentration fields. Fusion techniques aim to integrate these complementary strengths. Unlike purely statistical interpolation or regression-based bias correction, the fusion approach used in this study is model-guided, meaning that the spatial structure of concentrations is determined by the CTM, while observational data are used to adjust magnitude.

Data Fusion Method and Implementation Optimization: *We developed and published a generalized, user-friendly data fusion method (Gen-Friberg: GF-1) to reduce differences between chemical transport models (CTMs) and observational data is implemented to be compatible with widely used CTMs such as CMAQ, GEOS-Chem, and WRF-Chem* ***(Li et al., 2026).*** GF-1 is an advancement of the Friberg et al. [29] approach, which includes three general steps to fuse observational and simulation data to reduce spatiotemporal biases of CTM outputs.

The fused concentration field in the first step is obtained using either a zero-intercept linear or an exponential equation, derived by comparing annual mean observational data with CTM data. The selected equation aims to minimize yearly bias. Then, Kriging is conducted to reduce the spatial bias in the simulation. Since the zero-intercept linear regression is a special case of exponential regression (the exponent is 1), we combine these two cases and mathematically express the first step as follows.

$$\overline{OBS_m} = \alpha \times \overline{CTM(x)_m}^{\beta} + \varepsilon \tag{1}$$

$$\overline{FC(x)} = \alpha_{year} \times \overline{CTM(x)}^{\beta} \tag{2}$$

$$FC_1(x,t) = \left(\frac{OBS_m(t)}{\overline{OBS_m}}\right)_{krig} \times \overline{FC(x)} \tag{3}$$

Equation 1 is a regression equation for adjusting the annual mean of CTM predictions, where $OBS_m(t)$ are the daily observations at monitor m on day t, $\overline{OBS_m}$ is the monitor's annual mean value, $\overline{CTM(x)_m}$ is the annual mean CTM concentrations at corresponding monitor locations. ε denotes the regression residual, minimized through the least squares method during the estimation of regression parameters α and β. $\overline{FC(x)}$ is the adjusted annual mean gridded CTM concentration ($\overline{CTM(x)}$) over the study domain for each grid cell at location x, preserving the annual spatial distributions from CTM with adjusted intensity. To adjust the annual mean gridded CTM values, we first derived the regression parameter β (note for linear regression $\beta = 1$) based on all averaged observations at different monitors and their co-located annual

average CTM simulations (Equation 1) over all studied years. Then, the regression parameters α_{year} are estimated based on annual averaged observations and their co-located annual average CTM simulations for each year. This regression method is expected to capture the linear (when $\beta = 1$) or non-linear relations between observations and corresponding co-located simulations. For exponential regression, the exponent β is derived from all annual average observations and CTM simulations at monitor locations for each year. The slope α_{year} is derived using the specific year's annual average observations and CTM data. Friberg et al.'s study did not propose a selection algorithm for linear or exponential regression. For GF-1, we implement both modes of regression (i.e., linear and exponential). An algorithm that uses 10-fold cross-validation for selecting the optimal regression is provided (default mode). The regression with the lowest root mean square error (RMSE) is automatically selected for CTM adjustment. The normalized observations are interpolated by the Kriging interpolation to derive the FC_1. We implemented the Kriging interpolation using the PyKrige package [39] with the exponential variogram model. Then, the interpolated normalized observations are multiplied by the annual intensity-adjusted CTM concentration field ($\overline{FC(x)}$) to reduce the spatial distribution difference between simulations and observations.

The second step of the data fusion method mainly focuses on reducing the seasonal bias in CTM simulation results. First, the daily CTM results are adjusted using Equation 4, where $CTM(x,t)$ is the daily CTM data. $\overline{FC(x)}$ and $\overline{CTM(x)}$ are the annual spatial averages as before.

$$CTM(x,t)_{adj} = CTM(x,t) \times \frac{\overline{FC(x)}}{\overline{CTM(x)}} \tag{4}$$

$$\overline{CTM_{adj}(t)} = \frac{\sum_{m=1}^{M} CTM_{adj}(m,t)}{M} \tag{5}$$

$$\overline{OBS(t)} = \frac{\sum_{m=1}^{M} OBS_m(t)}{M} \tag{6}$$

The seasonal ratio β_{season}, which is the ratio of $\overline{CTM_{adj}(t)}$ to $\overline{OBS(t)}$ for each Julian day jt, is used to train the following trigonometric sinusoidal function:

$$\frac{\overline{CTM_{adj}(t)}}{\overline{OBS(t)}} = \beta_{season}(jt) = e^{A \times cos\left[\frac{2\pi}{365.25}(jt - jt_{max})\right]} + \varepsilon \tag{7}$$

The ratio captures the seasonal variation of both modeled and measured data sets. The period of the trigonometric function is 365.25 days. A and jt_{max} are parameters derived from the regression by minimizing the regression residual ε. jt is the Julian date of day t.

After adjusting the daily CTM results and developing the temporal regression function, the fused-concentration field in the second step (FC_2) is calculated as follows:

$$FC_2(x,t) = CTM(x,t)_{adj} \times \beta_{season}(jt) \tag{8}$$

In the third step, the method optimally integrates the results from the first and second steps to produce the final data fusion output. For grid cell location s, the method estimates the observation's correlations to FC_1 and FC_2 separately. Then, the method uses these correlations

to derive weights for calculating the weighted average of FC_1 and FC_2 as the final combined data fusion result. To calculate the weighting factor for FC_1, which has spatial information provided by interpolated observations, the method uses an exponential correlogram equation to estimate spatial correlations for each CTM grid cell. First, the method calculates the distance d and Pearson correlation value, R_{obs}, between each monitor pairing. Parameters R_{coll} and r are then determined based on these values using the exponential correlogram equation as follows:

$$R_{obs}(d) = R_{coll}e^{-\frac{d}{r}} + \varepsilon \tag{9}$$

R_{coll} is the intercept, which represents instrumentation error, while r is the distance at which the R_{obs} has an e-fold decrease. ε is the regression residual, which can be minimized by fitting given values of $R_{obs}(d)$ and their corresponding d. Then, for any CTM grid location denoted by x on day t, the correlation weighting factor $R_1(x,t)$ for FC_1 is estimated by:

$$R_1(s,t) = R_{coll}e^{-\frac{X(x,t)}{r}} \tag{10}$$

where $X(x,t)$ is the distance between grid cell location s and its closest monitor with data collected on day t. For FC_2, the spatial information is provided by the CTM. The method evaluates the CTM's performance in predicting spatial concentration distribution by calculating the mean Pearson correlation between the observation and CTM prediction at included monitors.

$$R_2 = \frac{1}{M}\sum_{m=1}^{M} corr(OBS_m, CTM_m) \tag{11}$$

where OBS_m is the time series of observational data at monitor m over the entire study period and the CTM_m is the time series of associated CTM predictions. Then, a combined weight factor is calculated as follows.

$$W(x,t) = \frac{R_1(x,t) \times (1 - R_2)}{R_1(x,t) \times (1 - R_2) + R_2 \times \left(1 - R_1(x,t)\right)} \tag{12}$$

The weight factor evaluates the confidence in using the FC_1 results at grid cell location x on day t. When the area is near the monitor, R_1 is dominant, leading to a higher weight W. It suggests placing more trust in FC_1, which includes the interpolated observations for spatial concentration distribution estimation. For locations far from the monitors, FC_2, which represents the CTM with seasonal correction, plays a dominant role in the data fusion. With the weight factor W, the method uses the weighted average on FC_1 and FC_2 to calculate the ultimate data fusion results FC_{opt}.

$$FC_{opt} = W(x,t) \times FC_1(x,t) + \left(1 - W(x,t)\right) \times FC_2(x,t) \tag{13}$$

Global regression parameters are calculated first (i.e., β, R_{coll}, r, A, and t_{max}), before proceeding with the steps of fusing the CTM simulation data with the corresponding yearly observation data set. After determining the parameters, the yearly data fusion processes for different years

were independent from each other, so it was implemented in parallel to speed up long-period data fusion.

Ratio-based fusion formulation: Total $PM_{2.5}$ and $PM_{2.5}$ chemical species (e.g., sulfate, nitrate, ammonium, organic carbon, elemental carbon) cannot be fused independently because doing so would violate mass balance between total $PM_{2.5}$ and its components. If total $PM_{2.5}$ and individual species were fused separately, the sum of the fused species would not necessarily equal the fused total $PM_{2.5}$ concentration ($\sum \mathrm{PM}_{2.5\text{-species}} \neq \mathrm{PM}_{2.5}$), leading to physically inconsistent aerosol composition. This mismatch would create physically unrealistic chemical composition and compromise subsequent interpretation of source contributions and health relevance. In addition, $PM_{2.5}$ species are typically measured once every three days at CSN and IMPROVE sites, whereas total $PM_{2.5}$ is measured daily. Independent species-level fusion would therefore introduce temporal inconsistencies and additional bias. To address these issues, we applied a ratio-based fusion approach. Specifically, CMAQ-simulated species-to-total $PM_{2.5}$ ratios were adjusted using observed species-to-total $PM_{2.5}$ ratios at monitoring sites. The fused species fractions were then multiplied by the fused total $PM_{2.5}$ concentration to obtain final species concentrations. This approach ensures that the sum of all fused species exactly equals the fused $PM_{2.5}$ mass, thereby preserving mass balance. By constraining chemical composition through fractional adjustment rather than absolute concentration fusion, this method retains physically consistent aerosol composition, minimizes bias from intermittent speciation sampling, and preserves the spatial structure of the CTM.

For each species s, the modeled ratio is defined as:

$$R_s^{\mathrm{model}}(x,t) = \frac{C_s^{\mathrm{model}}(x,t)}{C_{\mathrm{PM}_{2.5}}^{\mathrm{model}}(x,t)}$$

Similarly, an observed ratio is derived from collocated speciation measurements:

$$R_s^{\mathrm{obs}}(t) = \frac{C_s^{\mathrm{obs}}(t)}{C_{\mathrm{PM}_{2.5}}^{\mathrm{obs}}(t)}$$

These observed-to-modeled ratios are then spatially interpolated and fused, producing an adjusted species fraction field:

$$R_s^{\mathrm{fused}}(x,t)$$

Final fused species concentrations are calculated as:

$$C_s^{\mathrm{fused}}(x,t) = R_s^{\mathrm{fused}}(x,t) \times C_{\mathrm{PM}_{2.5}}^{\mathrm{fused}}(x,t)$$

This approach guarantees that:

$$\sum_s C_s^{\mathrm{fused}}(x,t) = C_{\mathrm{PM}_{2.5}}^{\mathrm{fused}}(x,t)$$

thereby explicitly preserving mass balance.

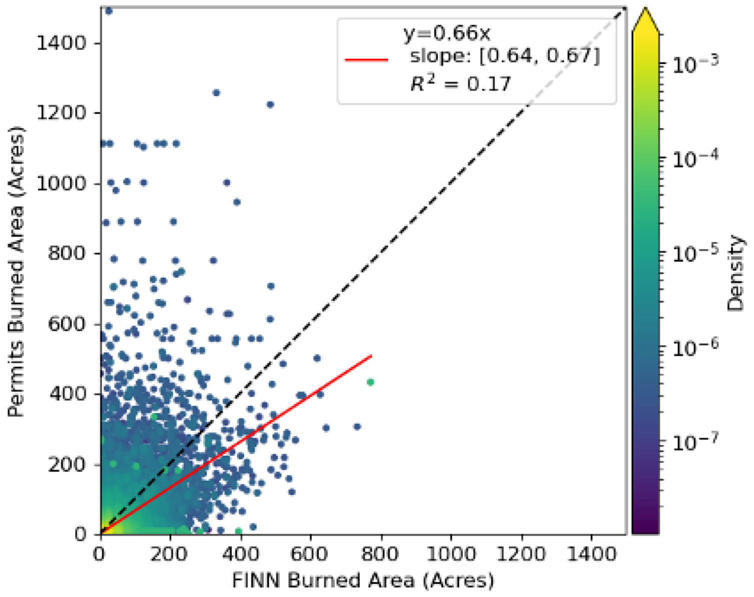

Figure S2: A linear regression between the FINN burned area and permit burn area recorded data (unit: acres) of prescribed burns matched over 4 km grid cells. The black line is a 1:1 line and the red line is the regression line (Li et al., 2023).

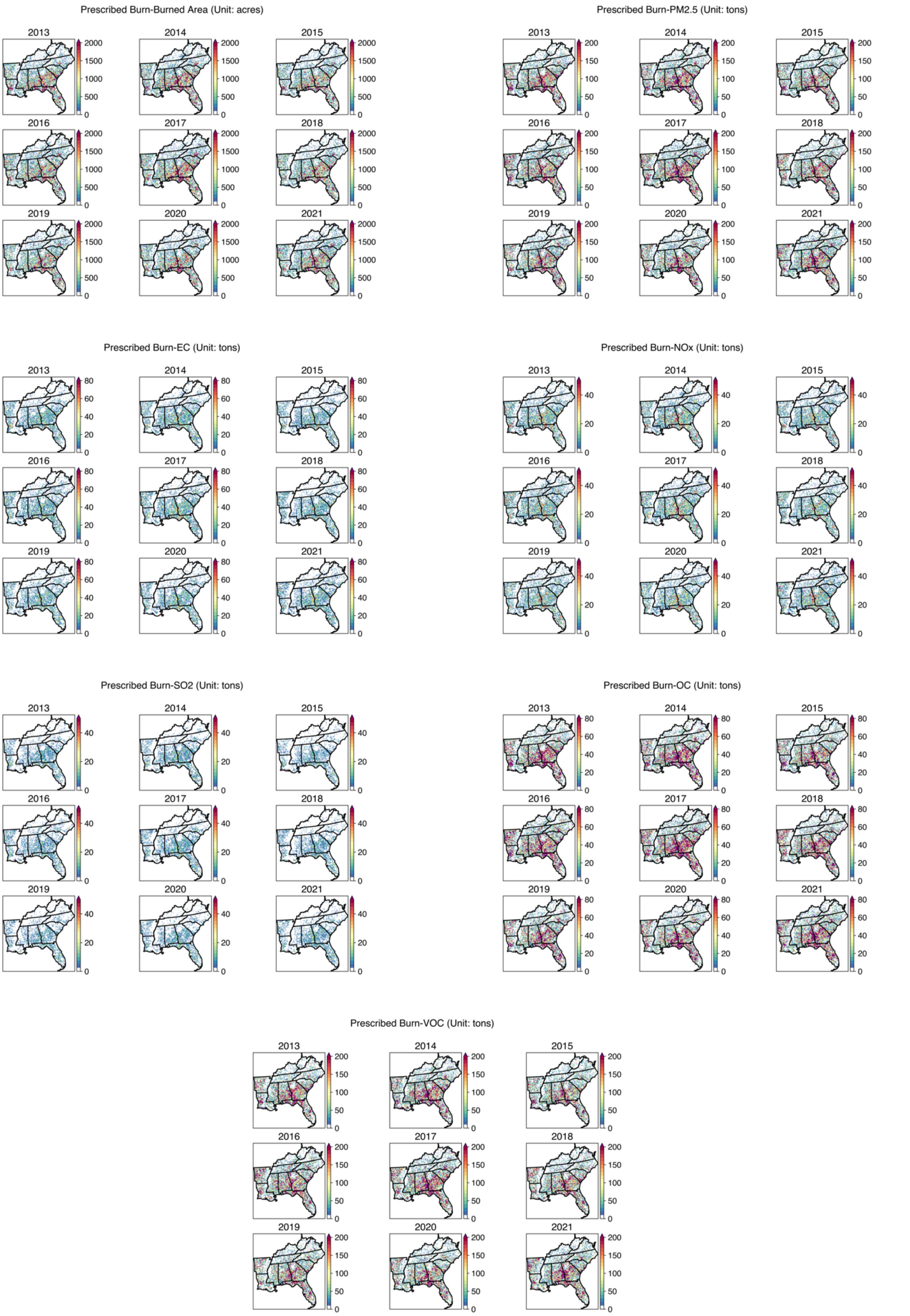

Figure S3: Spatial distribution of yearly prescribed burn area (unit: acres) and corresponding emission of $PM_{2.5}$, EC, OC, NOx, SO_2 and VOCs (unit: tons) from 2013-2021.

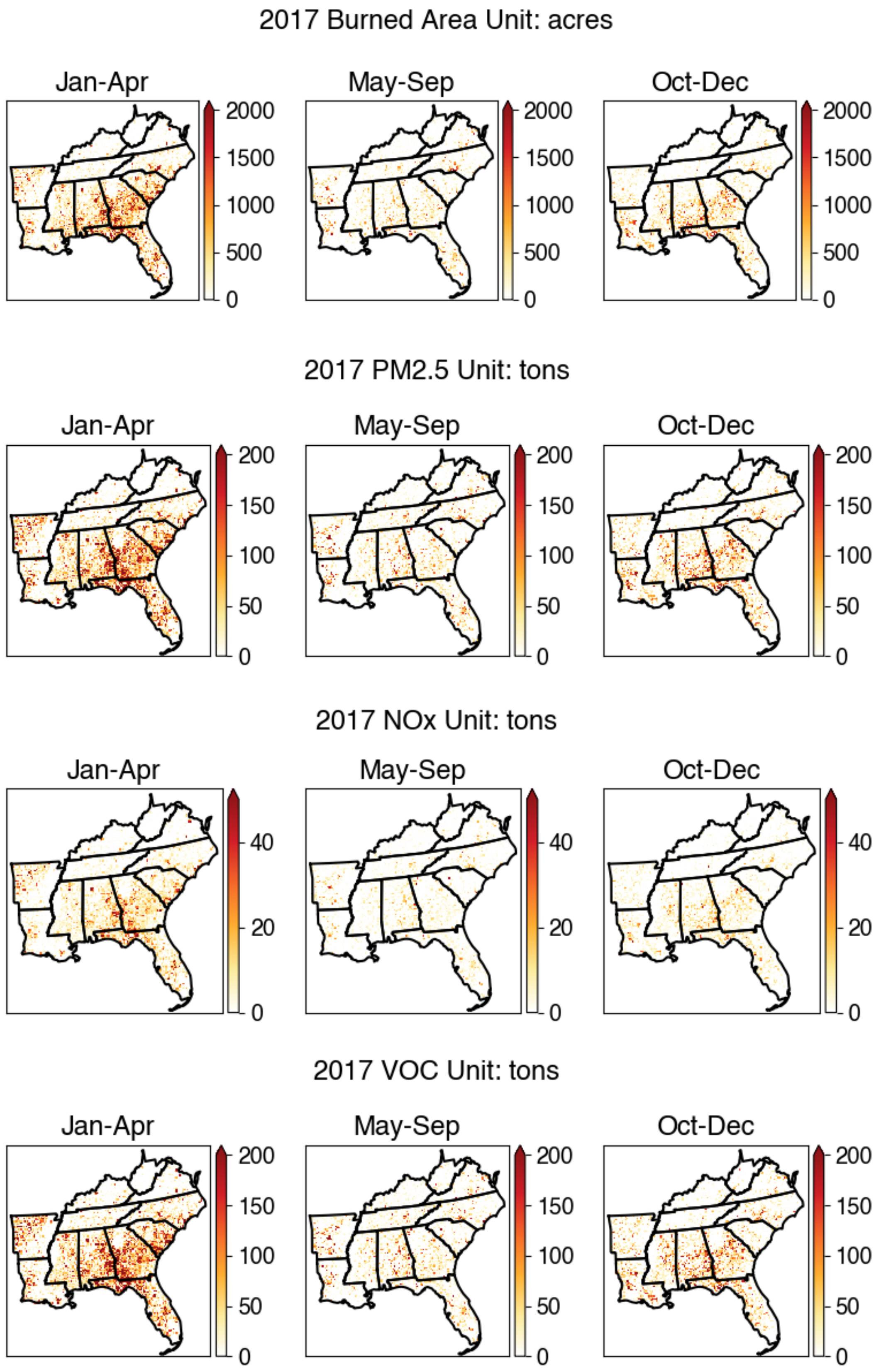

Figure S4: Spatial distribution of seasonal prescribed burn area (unit: acres) and corresponding emission of $PM_{2.5}$, NOx, and VOCs (unit: tons) in 2017.

Model vs Observation

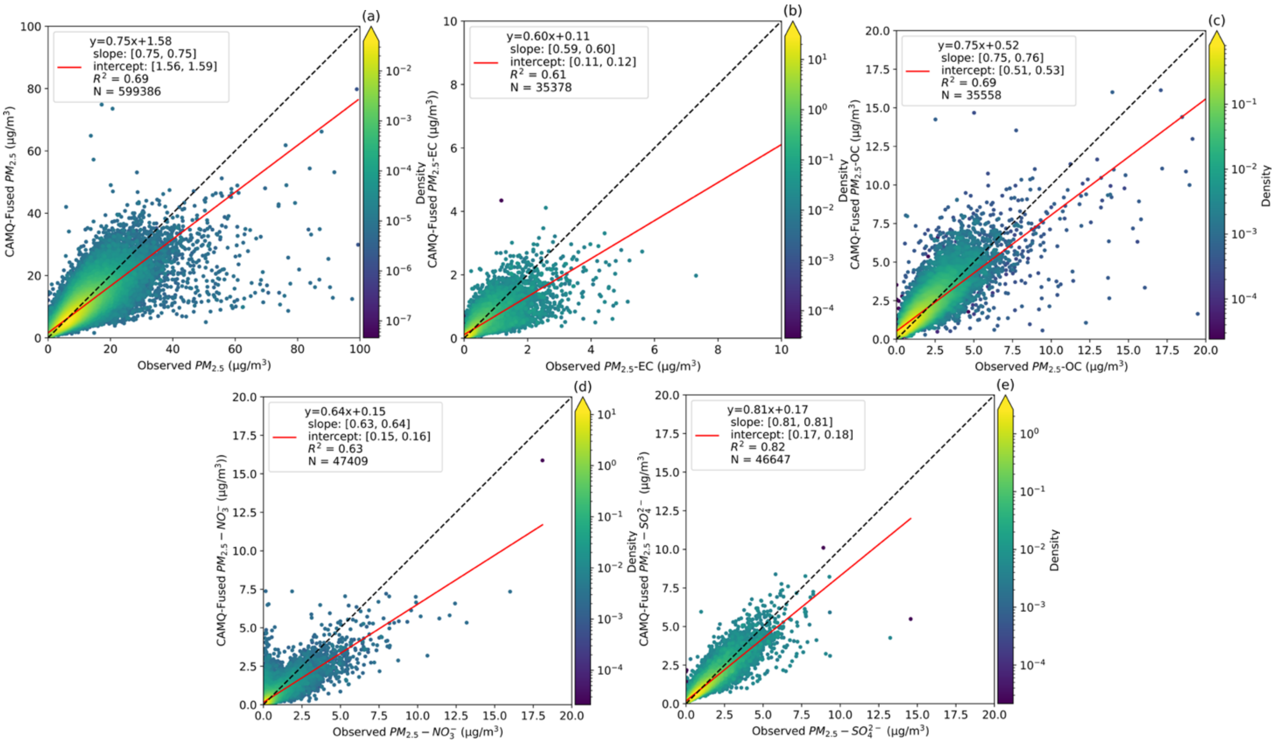

Figure S5: Density scatterplots of observed and data-fused daily average (a) PM2.5 and (b) EC, (c) OC, NO_3^- and (e) SO_4^{2-} during 2013–2021. The dotted line shows 1:1.

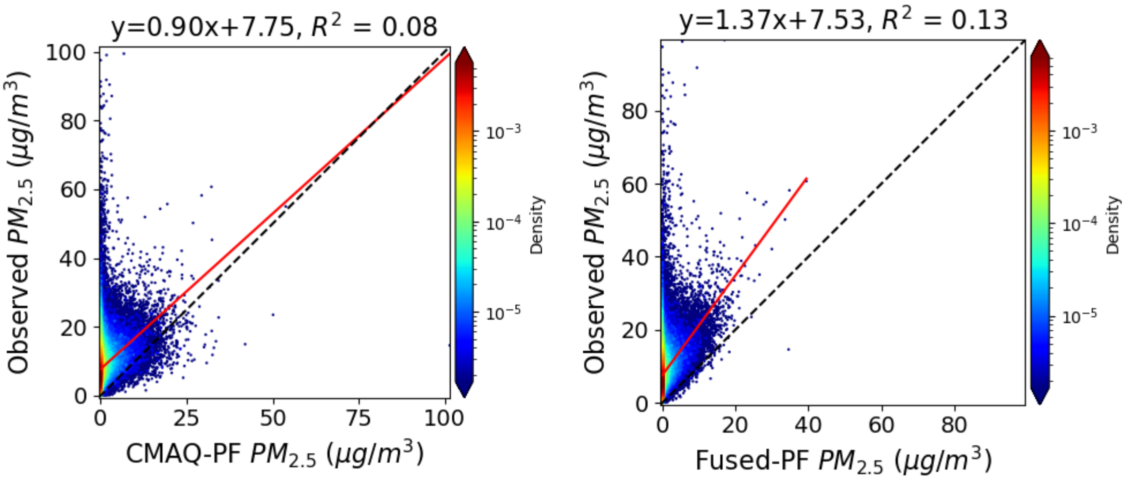

Figure S6: Observed PM2.5 versus estimated PF-PM2.5 from CMAQ (left) and data fusion (right).

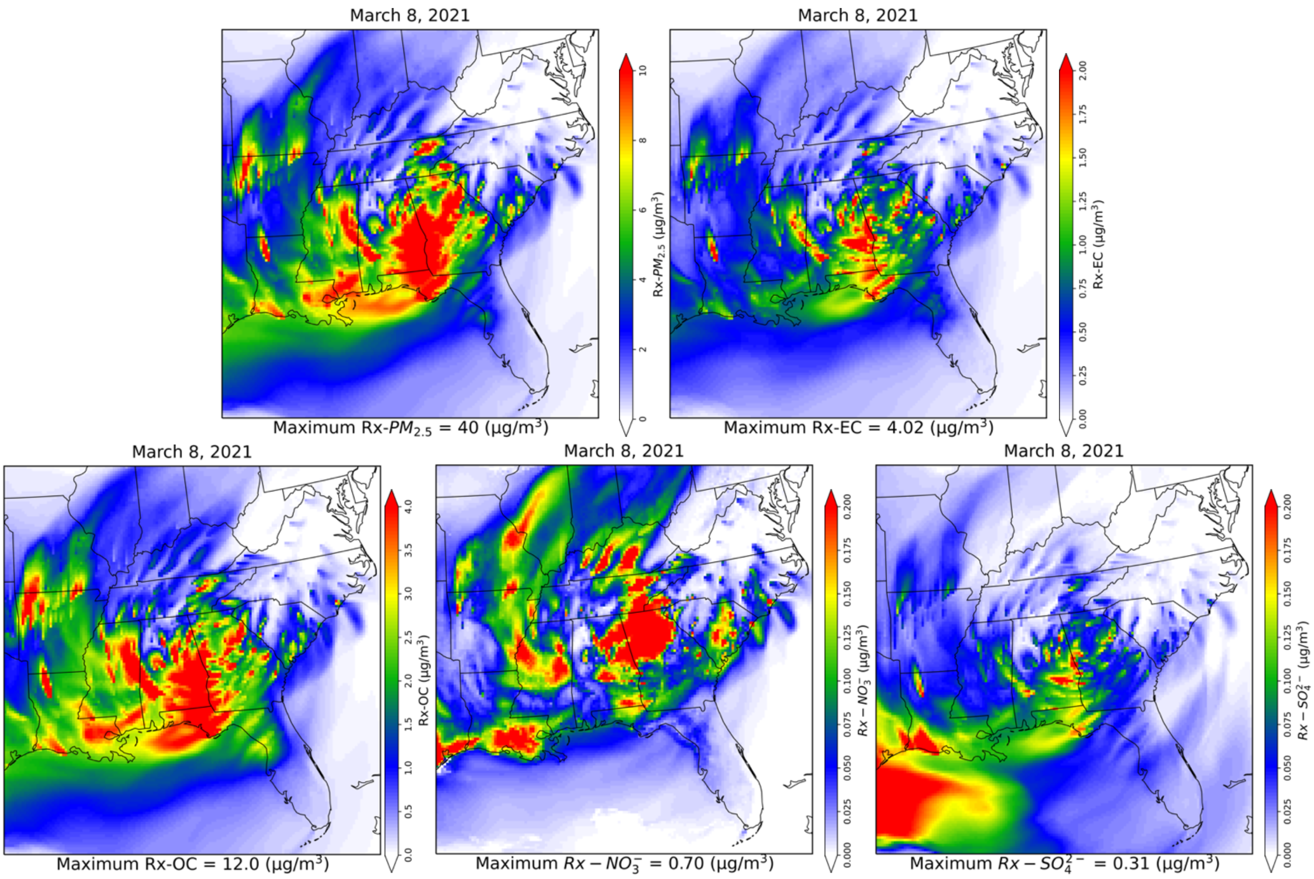

Figure S7: Prescribed burn $PM_{2.5}$, EC, OC, NO_3^- and SO_4^{2-} on March 8, 2021. The date was the highest burn area in 2021.

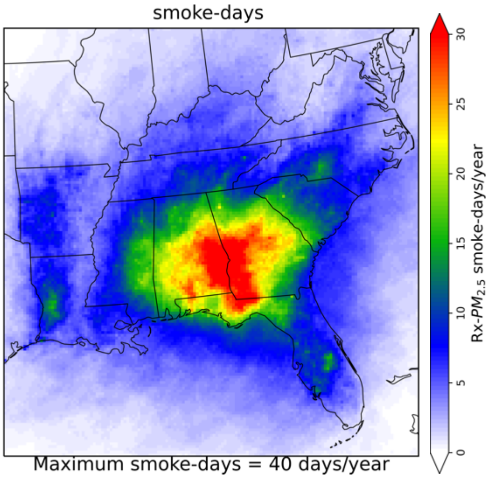

Figure S8: Distributions of the yearly smoke-days due to prescribed fire $PM_{2.5}$ during 2013–2021. Prescribed fire smoke-day as when prescribed burn $PM_{2.5}$ contributed ≥ 10% of proposed-NAAQS (35µg/m^3), i.e., ≥ 3.5 µg/m^3.

Table S1. CMAQ and data-fusion model performances with respect to EPA measurements in twelve southeastern US states during 2013-2021

	$PM_{2.5}$			EC			OC		
Measure	No fusion	Fusion	10-fold CV	No fusion	Fusion	10-fold CV	No fusion	Fusion	10-fold CV
MB	-1.27	-0.45	-0.56	-0.10	-0.05	-0.06	0.21	0.13	0.16
ME	3.14	1.66	2.23	0.20	0.14	0.16	0.81	0.43	0.52
RMSE	4.40	2.43	3.55	0.37	0.27	0.29	1.44	0.79	0.86
CRMSE	4.21	2.38	3.41	0.35	0.27	0.30	1.42	0.77	0.82
NMB	-15.51	-5.51	-7.63	-25.42	-12.03	-16.52	13.20	8.05	9.18
NME	38.28	20.22	24.81	49.58	34.34	37.59	51.19	27.10	30.20
MNB	-5.76	1.49	2.88	-6.61	8.41	6.26	41.95	27.45	32.66
MNE	43.01	23.63	29.49	56.02	41.03	46.11	74.32	40.51	50.92
FB	-21.63	-4.35	-8.82	-31.47	-6.34	-9.62	4.88	12.02	11.01
FE	43.69	21.32	27.21	57.92	34.66	37.83	48.65	27.28	32.67
R^2	0.29	0.69	0.62	0.36	0.61	0.57	0.30	0.69	0.64

	NO_3^-			SO_4^{2-}		
Measure	No fusion	Fusion	10-fold CV	No fusion	Fusion	10-fold CV
MB	-0.07	-0.01	-0.03	-0.28	-0.05	-0.11
ME	0.30	0.20	0.23	0.47	0.22	0.27
RMSE	0.54	0.39	0.42	0.69	0.35	0.41
CRMSE	0.54	0.39	0.42	0.63	0.35	0.41
NMB	-14.24	-3.05	-5.15	-23.67	-4.51	-7.29
NME	65.24	43.43	49.52	38.85	18.44	23.41
MNB	-10.30	30.63	27.44	-0.78	6.48	5.23
MNE	81.46	63.79	67.67	48.32	24.60	28.94
FB	-57.56	1.10	4.53	-21.54	-1.29	-6.35
FE	87.82	44.10	49.40	43.42	19.15	23.87
R^2	0.47	0.63	0.58	0.40	0.82	0.74

Mean bias (MB) (in concentration unit); Mean error (ME) (in concentration unit); Root mean square error (RMSE) (in concentration unit); Centered RMSE (CRMSE) (in concentration unit); Normalized mean bias (NMB) (in %); Normalized mean error (NME) (in %); Mean normalized bias (MNB) (in %); Mean normalized error (MNE) (in %); Fractional bias (FB) (in %); Fractional error (FE) (in %); coefficient of determination (R^2) (unitless); Cross Validation (CV)

Statistical measures for model performance evaluation discussed in this paper.

Mean bias (MB) $=\frac{1}{N}\sum(M_j - O_j)$

Mean error (ME) $=\frac{1}{N}\sum|M_j - O_j|$

Root means square error (RMSE) = $\sqrt{\frac{1}{N}\sum(M_j - O_j)^2}$

Root means square error (RMSE) = $\sqrt{\frac{1}{N}\sum[(M_j - \overline{M_j}) - (O_j - \bar{O}_j)]^2}$

Normalized mean bias (NMB) = $\frac{\sum(M_j - O_j)}{\sum O_j} \times 100$

Normalized mean error (NME) = $\frac{\sum|M_j - O_j|}{\sum O_j} \times 100$

Mean normalized bias (MNB) = $\frac{1}{N}\sum\frac{(M_j - O_j)}{O_j} \times 100$

Mean normalized error (MNE) = $\frac{1}{N}\sum\frac{|M_j - O_j|}{O_j} \times 100$

Fractional bias (FB) = $\frac{2}{N}\sum\frac{(M_j - O_j)}{(M_j + O_j)} \times 100$

Fractional error (FE) == $\frac{2}{N}\sum\frac{|M_j - O_j|}{|M_j + O_j|} \times 100$

Note. Subscript j represents the pairing of N observations O and Model predictions M by site and time. Overbars signify means over site and/or time.

Table S2: State-level yearly average prescribed burn smoke $PM_{2.5}$ concentrations (μg/m^3).

	Average 2013-2021	2013	2014	2015	2016	2017	2018	2019	2020	2021
Alabama	0.83±0.16 (0.84)	0.81±0.13 (0.82)	0.77±0.16 (0.78)	0.66±0.14 (0.68)	0.82±0.14 (0.83)	1.01±0.23 (0.98)	0.83±0.17 (0.81)	0.88±0.15 (0.88)	0.77±0.13 (0.77)	0.96±0.27 (0.9)
Arkansas	0.42±0.05 (0.41)	0.44±0.06 (0.44)	0.33±0.05 (0.33)	0.33±0.04 (0.33)	0.47±0.09 (0.47)	0.5±0.06 (0.48)	0.45±0.07 (0.44)	0.41±0.06 (0.41)	0.43±0.06 (0.43)	0.42±0.06 (0.4)
Florida	0.51±0.22 (0.52)	0.52±0.24 (0.49)	0.53±0.2 (0.56)	0.43±0.17 (0.43)	0.48±0.23 (0.47)	0.64±0.29 (0.63)	0.54±0.23 (0.54)	0.54±0.22 (0.53)	0.42±0.22 (0.42)	0.49±0.25 (0.49)
Georgia	0.85±0.17 (0.86)	0.77±0.17 (0.76)	0.88±0.18 (0.89)	0.6±0.14 (0.59)	0.84±0.15 (0.83)	1.13±0.24 (1.18)	0.85±0.15 (0.86)	0.86±0.16 (0.88)	0.71±0.17 (0.72)	1.04±0.24 (1.07)
Kentucky	0.36±0.04 (0.36)	0.36±0.04 (0.36)	0.29±0.04 (0.29)	0.28±0.03 (0.28)	0.39±0.06 (0.4)	0.44±0.05 (0.44)	0.34±0.05 (0.34)	0.36±0.06 (0.35)	0.34±0.04 (0.34)	0.43±0.06 (0.42)
Louisiana	0.44±0.06 (0.42)	0.45±0.08 (0.44)	0.42±0.1 (0.41)	0.38±0.07 (0.37)	0.46±0.08 (0.44)	0.47±0.08 (0.45)	0.43±0.06 (0.42)	0.47±0.06 (0.47)	0.44±0.07 (0.42)	0.4±0.06 (0.39)
Mississippi	0.56±0.1 (0.55)	0.57±0.09 (0.56)	0.49±0.11 (0.49)	0.47±0.09 (0.45)	0.56±0.1 (0.55)	0.67±0.13 (0.67)	0.55±0.11 (0.55)	0.63±0.1 (0.63)	0.53±0.1 (0.52)	0.52±0.11 (0.5)
North Carolina	0.42±0.1 (0.43)	0.32±0.08 (0.32)	0.41±0.1 (0.41)	0.3±0.08 (0.3)	0.48±0.1 (0.46)	0.6±0.15 (0.59)	0.4±0.11 (0.41)	0.43±0.12 (0.44)	0.35±0.07 (0.35)	0.55±0.13 (0.55)
South Carolina	0.6±0.09 (0.6)	0.51±0.1 (0.49)	0.64±0.09 (0.62)	0.41±0.07 (0.42)	0.59±0.08 (0.6)	0.87±0.13 (0.87)	0.6±0.08 (0.59)	0.58±0.1 (0.58)	0.43±0.06 (0.42)	0.76±0.12 (0.77)
Tennessee	0.48±0.06 (0.47)	0.47±0.06 (0.48)	0.4±0.07 (0.4)	0.37±0.04 (0.36)	0.49±0.08 (0.49)	0.58±0.07 (0.57)	0.48±0.08 (0.47)	0.52±0.09 (0.51)	0.44±0.06 (0.43)	0.55±0.1 (0.55)
Virginia	0.3±0.06 (0.3)	0.23±0.04 (0.23)	0.26±0.06 (0.26)	0.22±0.05 (0.22)	0.36±0.07 (0.37)	0.39±0.09 (0.38)	0.29±0.07 (0.28)	0.31±0.08 (0.31)	0.27±0.04 (0.27)	0.37±0.08 (0.37)
West Virginia	0.26±0.04 (0.25)	0.24±0.04 (0.24)	0.23±0.05 (0.23)	0.19±0.03 (0.19)	0.29±0.05 (0.27)	0.32±0.05 (0.32)	0.25±0.04 (0.26)	0.27±0.06 (0.26)	0.22±0.03 (0.22)	0.29±0.06 (0.29)

Table S3: State-level extensive burning season (January-April) (JFMA) average prescribed burn smoke $PM_{2.5}$ concentrations ($\mu g/m^3$).

	Average 2013-2021	2013	2014	2015	2016	2017	2018	2019	2020	2021
Alabama	1.11±0.27 (1.07)	1.06±0.26 (1.02)	0.84±0.24 (0.82)	0.66±0.25 (0.66)	1.1±0.23 (1.07)	1.5±0.44 (1.4)	1.14±0.32 (1.1)	1.08±0.24 (1.04)	0.7±0.16 (0.67)	1.31±0.46 (1.17)
Arkansas	0.46±0.08 (0.43)	0.4±0.08 (0.38)	0.3±0.07 (0.29)	0.2±0.06 (0.2)	0.48±0.15 (0.44)	0.51±0.1 (0.51)	0.62±0.13 (0.6)	0.41±0.1 (0.38)	0.23±0.05 (0.22)	0.43±0.12 (0.39)
Florida	0.88±0.32 (0.9)	1.03±0.42 (1)	0.57±0.24 (0.6)	0.75±0.21 (0.78)	0.73±0.33 (0.7)	1.02±0.45 (0.99)	1.09±0.45 (1.1)	0.8±0.3 (0.85)	0.59±0.26 (0.62)	0.79±0.36 (0.78)
Georgia	1.38±0.31 (1.38)	1.25±0.42 (1.23)	1.24±0.29 (1.25)	0.86±0.28 (0.83)	1.31±0.32 (1.29)	1.94±0.48 (2.01)	1.55±0.34 (1.56)	1.23±0.24 (1.26)	0.81±0.27 (0.75)	1.68±0.4 (1.72)
Kentucky	0.47±0.06 (0.46)	0.4±0.04 (0.4)	0.38±0.07 (0.37)	0.17±0.03 (0.16)	0.52±0.09 (0.51)	0.52±0.06 (0.52)	0.37±0.07 (0.35)	0.48±0.09 (0.46)	0.24±0.04 (0.23)	0.54±0.11 (0.54)
Louisiana	0.44±0.06 (0.44)	0.46±0.11 (0.44)	0.35±0.09 (0.35)	0.33±0.07 (0.33)	0.4±0.08 (0.38)	0.41±0.09 (0.41)	0.61±0.09 (0.61)	0.4±0.08 (0.38)	0.23±0.05 (0.23)	0.41±0.07 (0.4)
Mississippi	0.62±0.14 (0.62)	0.6±0.13 (0.57)	0.43±0.13 (0.39)	0.37±0.14 (0.33)	0.58±0.15 (0.57)	0.82±0.21 (0.81)	0.69±0.15 (0.69)	0.67±0.16 (0.68)	0.38±0.11 (0.37)	0.59±0.14 (0.58)
North Carolina	0.64±0.14 (0.64)	0.44±0.12 (0.43)	0.62±0.14 (0.63)	0.34±0.09 (0.34)	0.65±0.13 (0.64)	0.95±0.24 (0.92)	0.58±0.19 (0.61)	0.56±0.17 (0.58)	0.41±0.09 (0.4)	0.77±0.18 (0.76)
South Carolina	0.99±0.14 (0.97)	0.84±0.22 (0.79)	1.02±0.14 (1.01)	0.53±0.1 (0.51)	0.91±0.15 (0.89)	1.54±0.26 (1.52)	1.07±0.16 (1.05)	0.83±0.14 (0.82)	0.53±0.08 (0.53)	1.2±0.19 (1.18)
Tennessee	0.6±0.12 (0.6)	0.53±0.09 (0.52)	0.54±0.13 (0.55)	0.26±0.08 (0.25)	0.62±0.16 (0.64)	0.74±0.13 (0.73)	0.58±0.14 (0.56)	0.64±0.12 (0.64)	0.33±0.06 (0.33)	0.66±0.21 (0.62)
Virginia	0.42±0.07 (0.41)	0.24±0.06 (0.23)	0.34±0.09 (0.32)	0.2±0.05 (0.2)	0.51±0.08 (0.51)	0.56±0.11 (0.56)	0.35±0.1 (0.33)	0.4±0.11 (0.38)	0.23±0.06 (0.24)	0.45±0.1 (0.43)
West Virginia	0.35±0.05 (0.35)	0.25±0.04 (0.25)	0.29±0.06 (0.28)	0.16±0.03 (0.15)	0.41±0.06 (0.39)	0.41±0.06 (0.39)	0.3±0.05 (0.29)	0.36±0.09 (0.35)	0.15±0.03 (0.14)	0.39±0.08 (0.39)

Table S4: State-level low burning season (May-September) (MJJAS) average prescribed burn smoke $PM_{2.5}$ concentrations (μg/m^3).

	Average 2013-2021	2013	2014	2015	2016	2017	2018	2019	2020	2021
Alabama	0.54±0.07 (0.55)	0.51±0.09 (0.52)	0.47±0.09 (0.47)	0.52±0.08 (0.52)	0.59±0.12 (0.59)	0.45±0.08 (0.44)	0.58±0.1 (0.57)	0.67±0.1 (0.66)	0.41±0.09 (0.39)	0.48±0.11 (0.47)
Arkansas	0.41±0.07 (0.4)	0.48±0.09 (0.48)	0.31±0.07 (0.31)	0.42±0.07 (0.41)	0.37±0.08 (0.36)	0.34±0.09 (0.32)	0.42±0.08 (0.41)	0.39±0.07 (0.39)	0.31±0.07 (0.3)	0.33±0.07 (0.31)
Florida	0.3±0.14 (0.3)	0.21±0.13 (0.21)	0.28±0.12 (0.29)	0.25±0.14 (0.23)	0.28±0.15 (0.28)	0.3±0.16 (0.34)	0.25±0.15 (0.23)	0.34±0.18 (0.31)	0.24±0.12 (0.25)	0.17±0.12 (0.16)
Georgia	0.45±0.07 (0.46)	0.37±0.07 (0.38)	0.4±0.07 (0.41)	0.38±0.08 (0.39)	0.5±0.09 (0.52)	0.46±0.09 (0.47)	0.45±0.08 (0.44)	0.56±0.11 (0.54)	0.42±0.08 (0.42)	0.39±0.11 (0.41)
Kentucky	0.29±0.04 (0.29)	0.28±0.05 (0.27)	0.2±0.03 (0.2)	0.28±0.04 (0.27)	0.27±0.05 (0.27)	0.22±0.05 (0.21)	0.35±0.05 (0.35)	0.32±0.05 (0.32)	0.18±0.04 (0.18)	0.29±0.05 (0.29)
Louisiana	0.38±0.09 (0.37)	0.41±0.17 (0.37)	0.35±0.11 (0.35)	0.4±0.1 (0.39)	0.33±0.08 (0.33)	0.26±0.06 (0.25)	0.33±0.11 (0.32)	0.42±0.09 (0.42)	0.26±0.07 (0.24)	0.26±0.07 (0.25)
Mississippi	0.44±0.06 (0.43)	0.5±0.1 (0.51)	0.41±0.08 (0.39)	0.48±0.07 (0.46)	0.48±0.11 (0.46)	0.33±0.06 (0.32)	0.45±0.08 (0.44)	0.51±0.06 (0.51)	0.28±0.05 (0.26)	0.33±0.08 (0.31)
North Carolina	0.3±0.07 (0.29)	0.21±0.06 (0.2)	0.26±0.07 (0.25)	0.25±0.09 (0.25)	0.34±0.08 (0.33)	0.28±0.08 (0.29)	0.34±0.09 (0.33)	0.35±0.09 (0.35)	0.27±0.06 (0.25)	0.32±0.12 (0.29)
South Carolina	0.35±0.05 (0.35)	0.27±0.05 (0.27)	0.31±0.05 (0.33)	0.29±0.07 (0.28)	0.39±0.08 (0.39)	0.36±0.06 (0.36)	0.35±0.06 (0.34)	0.42±0.06 (0.42)	0.3±0.06 (0.3)	0.32±0.1 (0.32)
Tennessee	0.37±0.04 (0.37)	0.36±0.07 (0.36)	0.26±0.04 (0.25)	0.37±0.04 (0.37)	0.38±0.05 (0.38)	0.32±0.05 (0.32)	0.45±0.07 (0.45)	0.45±0.07 (0.44)	0.28±0.05 (0.27)	0.37±0.06 (0.37)
Virginia	0.25±0.05 (0.25)	0.18±0.05 (0.17)	0.18±0.05 (0.18)	0.22±0.05 (0.22)	0.25±0.06 (0.25)	0.2±0.07 (0.19)	0.32±0.08 (0.31)	0.29±0.07 (0.28)	0.21±0.05 (0.21)	0.28±0.07 (0.28)
West Virginia	0.21±0.04 (0.21)	0.18±0.05 (0.19)	0.17±0.04 (0.17)	0.2±0.04 (0.19)	0.19±0.06 (0.18)	0.13±0.04 (0.12)	0.29±0.05 (0.29)	0.24±0.05 (0.24)	0.15±0.03 (0.15)	0.19±0.05 (0.18)

Table S5: State-level moderate burning season (October-November) (OND) average prescribed burn smoke $PM_{2.5}$ concentrations (μg/m^3).

	Average 2013-2021	2013	2014	2015	2016	2017	2018	2019	2020	2021
Alabama	1.13±0.18 (1.15)	0.98±0.15 (1)	1.16±0.31 (1.15)	0.89±0.18 (0.91)	0.79±0.13 (0.78)	1.29±0.26 (1.28)	0.85±0.18 (0.86)	0.99±0.18 (1.01)	1.45±0.22 (1.48)	1.32±0.32 (1.25)
Arkansas	0.58±0.09 (0.58)	0.42±0.07 (0.41)	0.4±0.09 (0.4)	0.34±0.07 (0.36)	0.63±0.15 (0.61)	0.73±0.08 (0.73)	0.25±0.07 (0.24)	0.46±0.13 (0.44)	0.89±0.16 (0.89)	0.53±0.13 (0.54)
Florida	0.56±0.26 (0.52)	0.39±0.23 (0.35)	0.9±0.32 (0.94)	0.31±0.23 (0.26)	0.48±0.23 (0.47)	0.69±0.37 (0.62)	0.3±0.17 (0.29)	0.51±0.24 (0.46)	0.49±0.39 (0.4)	0.62±0.35 (0.58)
Georgia	0.97±0.2 (0.99)	0.8±0.24 (0.78)	1.2±0.31 (1.19)	0.64±0.15 (0.65)	0.73±0.13 (0.72)	1.2±0.28 (1.23)	0.61±0.21 (0.59)	0.87±0.23 (0.87)	1.07±0.3 (1.1)	1.26±0.29 (1.28)
Kentucky	0.52±0.06 (0.53)	0.44±0.07 (0.45)	0.32±0.04 (0.32)	0.42±0.06 (0.42)	0.42±0.06 (0.42)	0.7±0.07 (0.69)	0.29±0.05 (0.3)	0.28±0.05 (0.28)	0.74±0.11 (0.78)	0.53±0.08 (0.54)
Louisiana	0.7±0.09 (0.68)	0.51±0.11 (0.49)	0.65±0.2 (0.65)	0.43±0.09 (0.41)	0.74±0.14 (0.73)	0.87±0.2 (0.82)	0.36±0.09 (0.36)	0.66±0.09 (0.65)	0.99±0.19 (0.97)	0.63±0.11 (0.61)
Mississippi	0.82±0.14 (0.81)	0.67±0.16 (0.64)	0.73±0.2 (0.73)	0.57±0.14 (0.52)	0.66±0.1 (0.64)	1.05±0.19 (1.02)	0.55±0.17 (0.53)	0.79±0.13 (0.79)	1.17±0.19 (1.14)	0.76±0.15 (0.75)
North Carolina	0.47±0.13 (0.47)	0.34±0.11 (0.32)	0.38±0.14 (0.36)	0.32±0.09 (0.33)	0.47±0.14 (0.46)	0.65±0.23 (0.67)	0.26±0.11 (0.24)	0.41±0.13 (0.42)	0.42±0.15 (0.42)	0.64±0.16 (0.66)
South Carolina	0.62±0.11 (0.62)	0.48±0.14 (0.47)	0.68±0.18 (0.64)	0.43±0.1 (0.46)	0.52±0.08 (0.5)	0.86±0.17 (0.89)	0.4±0.13 (0.39)	0.52±0.16 (0.47)	0.51±0.11 (0.5)	0.93±0.14 (0.91)
Tennessee	0.65±0.09 (0.64)	0.58±0.1 (0.57)	0.45±0.07 (0.44)	0.5±0.09 (0.5)	0.51±0.07 (0.51)	0.8±0.09 (0.79)	0.4±0.08 (0.39)	0.49±0.11 (0.48)	0.85±0.16 (0.9)	0.71±0.11 (0.69)
Virginia	0.36±0.07 (0.36)	0.31±0.05 (0.31)	0.29±0.08 (0.29)	0.26±0.05 (0.26)	0.36±0.09 (0.37)	0.47±0.16 (0.45)	0.15±0.04 (0.14)	0.23±0.08 (0.21)	0.4±0.07 (0.42)	0.43±0.1 (0.44)
West Virginia	0.35±0.05 (0.35)	0.32±0.04 (0.31)	0.26±0.05 (0.25)	0.24±0.05 (0.24)	0.3±0.06 (0.28)	0.53±0.08 (0.54)	0.15±0.03 (0.15)	0.2±0.05 (0.19)	0.45±0.06 (0.44)	0.33±0.07 (0.33)

Table S6: State-level yearly average prescribed burn smoke EC concentrations (μg/m^3).

	Average 2013-2021	2013	2014	2015	2016	2017	2018	2019	2020	2021
Alabama	0.12±0.03 (0.12)	0.03±0.01 (0.03)	0.09±0.02 (0.09)	0.08±0.02 (0.08)	0.09±0.02 (0.1)	0.13±0.03 (0.13)	0.12±0.03 (0.12)	0.16±0.04 (0.15)	0.14±0.03 (0.14)	0.17±0.05 (0.15)
Arkansas	0.06±0.01 (0.06)	0.08±0.03 (0.08)	0.04±0.01 (0.04)	0.04±0.01 (0.04)	0.06±0.01 (0.06)	0.07±0.01 (0.07)	0.06±0.01 (0.06)	0.07±0.01 (0.07)	0.08±0.01 (0.08)	0.07±0.01 (0.07)
Florida	0.08±0.03 (0.08)	0.05±0.01 (0.04)	0.07±0.02 (0.07)	0.05±0.02 (0.05)	0.06±0.03 (0.06)	0.1±0.04 (0.1)	0.09±0.04 (0.09)	0.11±0.05 (0.11)	0.09±0.05 (0.08)	0.09±0.05 (0.09)
Georgia	0.12±0.03 (0.12)	0.07±0.01 (0.07)	0.1±0.02 (0.1)	0.07±0.02 (0.07)	0.1±0.02 (0.1)	0.15±0.04 (0.15)	0.12±0.03 (0.12)	0.16±0.03 (0.16)	0.13±0.04 (0.13)	0.17±0.04 (0.17)
Kentucky	0.05±0.01 (0.05)	0.1±0.02 (0.1)	0.03±0 (0.03)	0.03±0 (0.03)	0.04±0.01 (0.04)	0.06±0.01 (0.06)	0.04±0.01 (0.04)	0.06±0.01 (0.06)	0.06±0.01 (0.06)	0.07±0.01 (0.07)
Louisiana	0.07±0.01 (0.06)	0.11±0.02 (0.11)	0.05±0.01 (0.05)	0.05±0.01 (0.04)	0.05±0.01 (0.05)	0.07±0.02 (0.06)	0.06±0.01 (0.06)	0.09±0.01 (0.08)	0.08±0.02 (0.08)	0.07±0.02 (0.07)
Mississippi	0.08±0.02 (0.08)	0.07±0.02 (0.07)	0.06±0.01 (0.06)	0.05±0.01 (0.05)	0.06±0.01 (0.06)	0.09±0.02 (0.09)	0.08±0.02 (0.07)	0.11±0.02 (0.11)	0.1±0.02 (0.1)	0.09±0.02 (0.08)
North Carolina	0.06±0.01 (0.06)	0.06±0.01 (0.06)	0.04±0.01 (0.04)	0.03±0.01 (0.03)	0.05±0.01 (0.05)	0.07±0.02 (0.07)	0.05±0.02 (0.06)	0.07±0.02 (0.08)	0.06±0.01 (0.06)	0.09±0.02 (0.09)
South Carolina	0.08±0.01 (0.08)	0.04±0.01 (0.04)	0.07±0.01 (0.07)	0.04±0.01 (0.04)	0.07±0.01 (0.07)	0.1±0.02 (0.1)	0.08±0.01 (0.08)	0.1±0.02 (0.1)	0.07±0.01 (0.07)	0.12±0.02 (0.12)
Tennessee	0.07±0.01 (0.06)	0.06±0.01 (0.06)	0.04±0.01 (0.04)	0.04±0.01 (0.04)	0.06±0.01 (0.06)	0.07±0.01 (0.07)	0.06±0.01 (0.06)	0.09±0.02 (0.08)	0.07±0.01 (0.08)	0.09±0.02 (0.09)
Virginia	0.04±0.01 (0.04)	0.08±0.01 (0.08)	0.03±0.01 (0.03)	0.02±0.01 (0.02)	0.04±0.01 (0.04)	0.05±0.01 (0.05)	0.04±0.01 (0.04)	0.05±0.01 (0.05)	0.04±0.01 (0.04)	0.06±0.01 (0.06)
West Virginia	0.03±0.01 (0.03)	0.03±0.01 (0.03)	0.02±0.01 (0.02)	0.02±0 (0.02)	0.03±0.01 (0.03)	0.04±0.01 (0.04)	0.03±0.01 (0.03)	0.04±0.01 (0.04)	0.04±0.01 (0.04)	0.04±0.01 (0.04)

Table S7: State-level extensive burning season (January-April) (JFMA) average prescribed burn smoke EC concentrations (μg/m^3).

	Average 2013-2021	2013	2014	2015	2016	2017	2018	2019	2020	2021
Alabama	0.14±0.04 (0.13)	0.13±0.04 (0.13)	0.1±0.03 (0.09)	0.07±0.03 (0.07)	0.14±0.03 (0.13)	0.17±0.06 (0.15)	0.16±0.05 (0.14)	0.21±0.06 (0.2)	0.12±0.04 (0.11)	0.2±0.07 (0.18)
Arkansas	0.06±0.02 (0.05)	0.06±0.02 (0.06)	0.04±0.01 (0.04)	0.02±0.01 (0.02)	0.07±0.03 (0.06)	0.06±0.02 (0.06)	0.09±0.03 (0.08)	0.08±0.03 (0.07)	0.04±0.01 (0.04)	0.07±0.03 (0.06)
Florida	0.13±0.05 (0.13)	0.16±0.06 (0.15)	0.07±0.03 (0.07)	0.09±0.03 (0.09)	0.11±0.05 (0.1)	0.14±0.07 (0.14)	0.17±0.07 (0.17)	0.18±0.07 (0.18)	0.12±0.06 (0.12)	0.14±0.07 (0.14)
Georgia	0.18±0.05 (0.19)	0.16±0.06 (0.16)	0.15±0.04 (0.15)	0.1±0.03 (0.09)	0.17±0.05 (0.17)	0.23±0.06 (0.24)	0.22±0.06 (0.22)	0.24±0.05 (0.24)	0.14±0.06 (0.13)	0.25±0.05 (0.25)
Kentucky	0.05±0.01 (0.05)	0.05±0.01 (0.05)	0.05±0.01 (0.05)	0.02±0 (0.02)	0.06±0.01 (0.06)	0.05±0.01 (0.05)	0.05±0.01 (0.05)	0.09±0.02 (0.08)	0.04±0.01 (0.04)	0.09±0.02 (0.09)
Louisiana	0.06±0.01 (0.06)	0.07±0.02 (0.07)	0.04±0.01 (0.04)	0.04±0.01 (0.04)	0.05±0.01 (0.05)	0.05±0.01 (0.05)	0.09±0.02 (0.09)	0.08±0.02 (0.08)	0.04±0.01 (0.04)	0.07±0.02 (0.06)
Mississippi	0.08±0.02 (0.08)	0.08±0.02 (0.08)	0.05±0.02 (0.04)	0.04±0.02 (0.04)	0.07±0.02 (0.07)	0.09±0.02 (0.09)	0.09±0.02 (0.09)	0.12±0.03 (0.12)	0.06±0.02 (0.06)	0.09±0.02 (0.09)
North Carolina	0.08±0.02 (0.08)	0.06±0.02 (0.06)	0.07±0.02 (0.07)	0.04±0.01 (0.04)	0.08±0.02 (0.08)	0.11±0.03 (0.1)	0.08±0.02 (0.08)	0.1±0.03 (0.11)	0.07±0.02 (0.07)	0.12±0.03 (0.12)
South Carolina	0.13±0.02 (0.13)	0.12±0.04 (0.11)	0.12±0.02 (0.12)	0.06±0.01 (0.05)	0.12±0.02 (0.12)	0.18±0.03 (0.17)	0.15±0.03 (0.14)	0.16±0.03 (0.16)	0.09±0.02 (0.08)	0.18±0.03 (0.18)
Tennessee	0.07±0.01 (0.07)	0.07±0.01 (0.07)	0.07±0.01 (0.06)	0.03±0.01 (0.03)	0.08±0.02 (0.08)	0.08±0.01 (0.08)	0.07±0.02 (0.07)	0.12±0.02 (0.12)	0.05±0.01 (0.05)	0.11±0.03 (0.1)
Virginia	0.05±0.01 (0.05)	0.03±0.01 (0.03)	0.04±0.01 (0.04)	0.02±0.01 (0.02)	0.07±0.01 (0.06)	0.06±0.01 (0.06)	0.05±0.01 (0.05)	0.07±0.02 (0.07)	0.04±0.01 (0.04)	0.07±0.02 (0.07)
West Virginia	0.04±0.01 (0.04)	0.03±0.01 (0.03)	0.04±0.01 (0.03)	0.02±0 (0.01)	0.05±0.01 (0.05)	0.04±0.01 (0.04)	0.04±0.01 (0.04)	0.07±0.02 (0.07)	0.02±0.01 (0.02)	0.06±0.01 (0.06)

Table S8: State-level low burning season (May-September) (MJJAS) average prescribed burn smoke EC concentrations (µg/m^3).

	Average 2013-2021	2013	2014	2015	2016	2017	2018	2019	2020	2021
Alabama	0.07±0.01 (0.07)	0.06±0.01 (0.06)	0.05±0.01 (0.05)	0.06±0.01 (0.06)	0.06±0.02 (0.06)	0.06±0.01 (0.06)	0.08±0.02 (0.07)	0.09±0.02 (0.09)	0.07±0.02 (0.06)	0.09±0.03 (0.08)
Arkansas	0.05±0.01 (0.05)	0.05±0.01 (0.05)	0.03±0.01 (0.03)	0.05±0.01 (0.04)	0.04±0.01 (0.04)	0.05±0.02 (0.04)	0.05±0.02 (0.05)	0.05±0.01 (0.05)	0.05±0.02 (0.05)	0.05±0.02 (0.05)
Florida	0.04±0.02 (0.04)	0.03±0.02 (0.03)	0.03±0.01 (0.03)	0.03±0.02 (0.03)	0.04±0.02 (0.04)	0.05±0.02 (0.05)	0.04±0.02 (0.04)	0.06±0.03 (0.05)	0.05±0.03 (0.05)	0.04±0.03 (0.03)
Georgia	0.05±0.01 (0.05)	0.04±0.01 (0.04)	0.04±0.01 (0.04)	0.04±0.01 (0.04)	0.05±0.01 (0.05)	0.06±0.02 (0.06)	0.06±0.01 (0.06)	0.07±0.02 (0.07)	0.07±0.02 (0.07)	0.07±0.02 (0.07)
Kentucky	0.03±0 (0.03)	0.03±0 (0.03)	0.02±0 (0.02)	0.03±0 (0.03)	0.02±0.01 (0.02)	0.03±0.01 (0.02)	0.04±0.01 (0.04)	0.04±0.01 (0.04)	0.03±0.01 (0.03)	0.04±0.01 (0.04)
Louisiana	0.05±0.01 (0.04)	0.05±0.02 (0.05)	0.04±0.01 (0.03)	0.05±0.02 (0.04)	0.03±0.01 (0.03)	0.04±0.01 (0.04)	0.04±0.02 (0.04)	0.06±0.02 (0.06)	0.05±0.02 (0.04)	0.05±0.02 (0.04)
Mississippi	0.05±0.01 (0.05)	0.06±0.01 (0.06)	0.04±0.01 (0.04)	0.06±0.01 (0.05)	0.05±0.01 (0.04)	0.04±0.01 (0.04)	0.05±0.01 (0.05)	0.07±0.01 (0.07)	0.05±0.01 (0.05)	0.06±0.02 (0.05)
North Carolina	0.03±0.01 (0.03)	0.03±0.01 (0.02)	0.02±0.01 (0.02)	0.03±0.01 (0.02)	0.03±0.01 (0.03)	0.03±0.01 (0.03)	0.04±0.02 (0.04)	0.05±0.02 (0.04)	0.04±0.02 (0.04)	0.05±0.02 (0.04)
South Carolina	0.04±0.01 (0.04)	0.03±0.01 (0.03)	0.03±0 (0.03)	0.03±0.01 (0.03)	0.03±0.01 (0.03)	0.04±0.01 (0.04)	0.04±0.01 (0.04)	0.05±0.01 (0.05)	0.04±0.01 (0.04)	0.05±0.02 (0.05)
Tennessee	0.04±0.01 (0.04)	0.04±0.01 (0.04)	0.03±0.01 (0.02)	0.04±0.01 (0.04)	0.04±0.01 (0.04)	0.04±0.01 (0.04)	0.05±0.01 (0.05)	0.06±0.01 (0.06)	0.04±0.01 (0.04)	0.06±0.01 (0.05)
Virginia	0.03±0.01 (0.03)	0.02±0.01 (0.02)	0.02±0.01 (0.01)	0.02±0.01 (0.02)	0.02±0.01 (0.02)	0.02±0.01 (0.02)	0.04±0.01 (0.03)	0.04±0.01 (0.03)	0.03±0.01 (0.03)	0.04±0.01 (0.04)
West Virginia	0.02±0.01 (0.02)	0.02±0.01 (0.02)	0.02±0.01 (0.01)	0.02±0 (0.02)	0.02±0.01 (0.02)	0.02±0.01 (0.01)	0.03±0.01 (0.03)	0.03±0.01 (0.03)	0.02±0.01 (0.02)	0.03±0.01 (0.02)

Table S9: State-level moderate burning season (October-November) (OND) average prescribed burn smoke EC concentrations (μg/m^3).

	Average 2013-2021	2013	2014	2015	2016	2017	2018	2019	2020	2021
Alabama	0.18±0.04 (0.18)	0.18±0.04 (0.17)	0.14±0.05 (0.14)	0.11±0.03 (0.11)	0.09±0.02 (0.09)	0.21±0.05 (0.19)	0.16±0.05 (0.16)	0.2±0.04 (0.19)	0.29±0.07 (0.29)	0.26±0.08 (0.24)
Arkansas	0.09±0.01 (0.09)	0.08±0.02 (0.08)	0.05±0.01 (0.05)	0.04±0.01 (0.04)	0.07±0.02 (0.07)	0.12±0.02 (0.11)	0.05±0.01 (0.04)	0.09±0.03 (0.08)	0.18±0.04 (0.18)	0.1±0.02 (0.1)
Florida	0.09±0.05 (0.08)	0.07±0.04 (0.06)	0.11±0.04 (0.11)	0.04±0.03 (0.03)	0.06±0.03 (0.06)	0.12±0.07 (0.11)	0.06±0.03 (0.05)	0.12±0.06 (0.1)	0.1±0.09 (0.08)	0.12±0.07 (0.11)
Georgia	0.15±0.04 (0.16)	0.14±0.04 (0.13)	0.14±0.04 (0.14)	0.08±0.02 (0.08)	0.08±0.02 (0.08)	0.19±0.05 (0.2)	0.11±0.05 (0.11)	0.18±0.06 (0.18)	0.23±0.08 (0.23)	0.24±0.07 (0.23)
Kentucky	0.07±0.01 (0.07)	0.07±0.01 (0.07)	0.03±0 (0.03)	0.05±0.01 (0.05)	0.05±0.01 (0.05)	0.11±0.02 (0.11)	0.05±0.01 (0.05)	0.05±0.01 (0.05)	0.13±0.03 (0.14)	0.09±0.02 (0.09)
Louisiana	0.11±0.02 (0.1)	0.1±0.03 (0.09)	0.08±0.02 (0.08)	0.05±0.01 (0.05)	0.08±0.02 (0.08)	0.14±0.04 (0.12)	0.06±0.02 (0.06)	0.14±0.03 (0.13)	0.19±0.04 (0.18)	0.12±0.03 (0.11)
Mississippi	0.13±0.03 (0.12)	0.13±0.03 (0.12)	0.09±0.03 (0.09)	0.07±0.02 (0.06)	0.07±0.01 (0.07)	0.17±0.04 (0.17)	0.1±0.04 (0.1)	0.15±0.03 (0.15)	0.23±0.04 (0.22)	0.14±0.03 (0.13)
North Carolina	0.07±0.02 (0.07)	0.06±0.02 (0.06)	0.04±0.02 (0.04)	0.03±0.01 (0.03)	0.06±0.02 (0.05)	0.1±0.04 (0.1)	0.05±0.02 (0.04)	0.08±0.03 (0.09)	0.08±0.04 (0.08)	0.12±0.04 (0.12)
South Carolina	0.09±0.02 (0.09)	0.08±0.02 (0.08)	0.07±0.02 (0.07)	0.05±0.01 (0.05)	0.05±0.01 (0.05)	0.12±0.02 (0.12)	0.07±0.03 (0.06)	0.1±0.04 (0.09)	0.09±0.02 (0.09)	0.17±0.03 (0.16)
Tennessee	0.09±0.01 (0.09)	0.09±0.02 (0.09)	0.05±0.01 (0.05)	0.06±0.01 (0.06)	0.06±0.01 (0.06)	0.13±0.02 (0.12)	0.07±0.02 (0.07)	0.1±0.02 (0.1)	0.16±0.04 (0.17)	0.13±0.02 (0.13)
Virginia	0.05±0.01 (0.05)	0.05±0.01 (0.05)	0.03±0.01 (0.03)	0.03±0.01 (0.03)	0.04±0.01 (0.04)	0.07±0.03 (0.07)	0.03±0.01 (0.02)	0.05±0.02 (0.04)	0.07±0.01 (0.07)	0.07±0.02 (0.07)
West Virginia	0.04±0.01 (0.04)	0.05±0.01 (0.05)	0.03±0.01 (0.02)	0.02±0.01 (0.02)	0.03±0.01 (0.03)	0.08±0.01 (0.08)	0.02±0 (0.02)	0.03±0.01 (0.03)	0.08±0.01 (0.08)	0.06±0.01 (0.06)

Table S10: State-level yearly average prescribed burn smoke OC concentrations (µg/m^3).

	Average 2013-2021	2013	2014	2015	2016	2017	2018	2019	2020	2021
Alabama	0.39±0.08 (0.39)	0.11±0.02 (0.11)	0.29±0.07 (0.29)	0.29±0.07 (0.29)	0.43±0.08 (0.43)	0.43±0.1 (0.42)	0.43±0.1 (0.42)	0.52±0.11 (0.52)	0.39±0.09 (0.38)	0.45±0.13 (0.42)
Arkansas	0.19±0.03 (0.19)	0.06±0.01 (0.06)	0.12±0.03 (0.12)	0.13±0.02 (0.13)	0.25±0.05 (0.24)	0.22±0.04 (0.21)	0.21±0.04 (0.2)	0.21±0.04 (0.21)	0.23±0.04 (0.22)	0.19±0.04 (0.18)
Florida	0.25±0.11 (0.24)	0.08±0.03 (0.07)	0.21±0.08 (0.22)	0.19±0.08 (0.18)	0.25±0.13 (0.24)	0.28±0.14 (0.27)	0.27±0.13 (0.26)	0.37±0.14 (0.37)	0.21±0.12 (0.2)	0.23±0.13 (0.22)
Georgia	0.40±0.08 (0.41)	0.1±0.02 (0.1)	0.33±0.08 (0.33)	0.26±0.07 (0.26)	0.43±0.08 (0.42)	0.48±0.1 (0.49)	0.42±0.08 (0.42)	0.54±0.12 (0.55)	0.36±0.09 (0.36)	0.46±0.11 (0.47)
Kentucky	0.15±0.02 (0.15)	0.04±0.01 (0.04)	0.09±0.02 (0.09)	0.11±0.01 (0.11)	0.19±0.03 (0.18)	0.18±0.02 (0.18)	0.15±0.02 (0.15)	0.17±0.03 (0.17)	0.16±0.02 (0.16)	0.18±0.03 (0.18)
Louisiana	0.21±0.03 (0.2)	0.06±0.01 (0.06)	0.15±0.02 (0.15)	0.17±0.03 (0.16)	0.24±0.05 (0.23)	0.2±0.04 (0.19)	0.21±0.04 (0.21)	0.28±0.04 (0.27)	0.22±0.04 (0.21)	0.19±0.04 (0.18)
Mississippi	0.26±0.05 (0.25)	0.08±0.01 (0.08)	0.18±0.04 (0.18)	0.2±0.05 (0.19)	0.29±0.05 (0.29)	0.29±0.06 (0.29)	0.28±0.06 (0.27)	0.36±0.07 (0.36)	0.27±0.05 (0.26)	0.24±0.06 (0.23)
North Carolina	0.19±0.05 (0.19)	0.04±0.01 (0.04)	0.14±0.04 (0.14)	0.12±0.03 (0.12)	0.23±0.06 (0.23)	0.24±0.06 (0.24)	0.18±0.06 (0.18)	0.24±0.07 (0.25)	0.17±0.04 (0.16)	0.25±0.06 (0.25)
South Carolina	0.27±0.04 (0.26)	0.07±0.02 (0.07)	0.23±0.04 (0.22)	0.17±0.03 (0.17)	0.29±0.04 (0.3)	0.35±0.05 (0.35)	0.28±0.04 (0.27)	0.34±0.07 (0.33)	0.2±0.04 (0.19)	0.34±0.06 (0.34)
Tennessee	0.21±0.03 (0.21)	0.06±0.01 (0.06)	0.13±0.02 (0.13)	0.15±0.02 (0.15)	0.24±0.04 (0.24)	0.25±0.03 (0.25)	0.22±0.04 (0.21)	0.27±0.05 (0.27)	0.21±0.03 (0.21)	0.25±0.05 (0.25)
Virginia	0.13±0.03 (0.13)	0.03±0.01 (0.03)	0.09±0.02 (0.09)	0.09±0.02 (0.09)	0.18±0.04 (0.18)	0.16±0.04 (0.16)	0.12±0.03 (0.12)	0.15±0.04 (0.15)	0.12±0.02 (0.12)	0.17±0.04 (0.16)
West Virginia	0.11±0.02 (0.1)	0.03±0.01 (0.03)	0.08±0.02 (0.07)	0.07±0.01 (0.07)	0.14±0.03 (0.13)	0.13±0.02 (0.13)	0.10±0.02 (0.11)	0.13±0.03 (0.12)	0.10±0.02 (0.1)	0.13±0.03 (0.12)

Table S11: State-level extensive burning season (January-April) (JFMA) average prescribed burn smoke OC concentrations (µg/m^3).

	Average 2013-2021	2013	2014	2015	2016	2017	2018	2019	2020	2021
Alabama	0.48±0.13 (0.46)	0.37±0.11 (0.35)	0.3±0.09 (0.3)	0.29±0.12 (0.29)	0.53±0.11 (0.51)	0.56±0.18 (0.52)	0.53±0.17 (0.49)	0.7±0.19 (0.68)	0.31±0.09 (0.29)	0.54±0.18 (0.49)
Arkansas	0.2±0.05 (0.19)	0.15±0.04 (0.14)	0.11±0.03 (0.11)	0.09±0.03 (0.09)	0.25±0.09 (0.22)	0.18±0.04 (0.18)	0.3±0.08 (0.28)	0.26±0.07 (0.24)	0.1±0.03 (0.09)	0.18±0.06 (0.16)
Florida	0.42±0.16 (0.43)	0.4±0.18 (0.38)	0.21±0.1 (0.21)	0.34±0.1 (0.35)	0.39±0.19 (0.37)	0.41±0.2 (0.39)	0.56±0.25 (0.53)	0.63±0.25 (0.66)	0.29±0.14 (0.29)	0.36±0.17 (0.36)
Georgia	0.62±0.14 (0.63)	0.46±0.16 (0.46)	0.47±0.11 (0.46)	0.39±0.13 (0.38)	0.67±0.16 (0.66)	0.76±0.19 (0.78)	0.74±0.18 (0.77)	0.86±0.19 (0.88)	0.36±0.14 (0.33)	0.67±0.14 (0.67)
Kentucky	0.19±0.03 (0.19)	0.12±0.02 (0.12)	0.14±0.03 (0.14)	0.07±0.02 (0.07)	0.23±0.05 (0.24)	0.18±0.03 (0.18)	0.16±0.03 (0.15)	0.27±0.06 (0.26)	0.09±0.02 (0.09)	0.22±0.05 (0.23)
Louisiana	0.2±0.03 (0.2)	0.17±0.04 (0.16)	0.12±0.03 (0.12)	0.15±0.04 (0.15)	0.2±0.05 (0.19)	0.15±0.04 (0.15)	0.31±0.06 (0.31)	0.27±0.07 (0.26)	0.11±0.03 (0.1)	0.18±0.04 (0.17)
Mississippi	0.26±0.06 (0.27)	0.21±0.05 (0.2)	0.15±0.05 (0.13)	0.17±0.07 (0.15)	0.28±0.07 (0.28)	0.29±0.08 (0.28)	0.31±0.06 (0.31)	0.42±0.1 (0.42)	0.15±0.05 (0.15)	0.23±0.06 (0.23)
North Carolina	0.28±0.06 (0.27)	0.16±0.05 (0.15)	0.23±0.05 (0.22)	0.14±0.04 (0.15)	0.31±0.06 (0.31)	0.35±0.09 (0.33)	0.25±0.08 (0.25)	0.36±0.12 (0.37)	0.18±0.05 (0.17)	0.33±0.08 (0.33)
South Carolina	0.44±0.07 (0.42)	0.31±0.09 (0.3)	0.38±0.05 (0.37)	0.23±0.05 (0.22)	0.46±0.09 (0.45)	0.57±0.1 (0.56)	0.49±0.1 (0.47)	0.59±0.12 (0.57)	0.22±0.04 (0.21)	0.51±0.08 (0.49)
Tennessee	0.25±0.05 (0.25)	0.17±0.03 (0.17)	0.19±0.05 (0.19)	0.11±0.04 (0.11)	0.29±0.08 (0.3)	0.27±0.05 (0.27)	0.25±0.06 (0.23)	0.37±0.07 (0.37)	0.13±0.03 (0.13)	0.28±0.09 (0.27)
Virginia	0.17±0.03 (0.17)	0.08±0.02 (0.07)	0.12±0.04 (0.11)	0.08±0.02 (0.08)	0.25±0.04 (0.24)	0.2±0.04 (0.19)	0.14±0.04 (0.14)	0.24±0.07 (0.24)	0.09±0.02 (0.09)	0.19±0.04 (0.18)
West Virginia	0.15±0.02 (0.14)	0.08±0.02 (0.08)	0.11±0.03 (0.1)	0.06±0.01 (0.06)	0.2±0.03 (0.19)	0.14±0.02 (0.14)	0.12±0.02 (0.12)	0.21±0.05 (0.21)	0.06±0.01 (0.05)	0.17±0.03 (0.16)

Table S12: State-level low burning season (May-September) (MJJAS) average prescribed burn smoke OC concentrations (μg/m^3).

	Average 2013-2021	2013	2014	2015	2016	2017	2018	2019	2020	2021
Alabama	0.24±0.04 (0.24)	0.21±0.05 (0.21)	0.14±0.03 (0.14)	0.21±0.04 (0.21)	0.31±0.11 (0.3)	0.24±0.05 (0.24)	0.26±0.06 (0.25)	0.21±0.04 (0.21)	0.2±0.06 (0.19)	0.26±0.08 (0.25)
Arkansas	0.17±0.04 (0.16)	0.18±0.05 (0.17)	0.1±0.03 (0.09)	0.16±0.04 (0.15)	0.18±0.06 (0.17)	0.18±0.06 (0.16)	0.17±0.05 (0.15)	0.11±0.03 (0.11)	0.15±0.05 (0.14)	0.17±0.05 (0.15)
Florida	0.12±0.06 (0.12)	0.08±0.05 (0.09)	0.08±0.04 (0.08)	0.1±0.06 (0.09)	0.12±0.08 (0.11)	0.16±0.09 (0.17)	0.1±0.06 (0.09)	0.1±0.06 (0.09)	0.12±0.06 (0.12)	0.09±0.07 (0.08)
Georgia	0.18±0.03 (0.18)	0.14±0.03 (0.14)	0.11±0.02 (0.11)	0.14±0.03 (0.14)	0.22±0.05 (0.23)	0.23±0.05 (0.23)	0.18±0.04 (0.17)	0.16±0.04 (0.15)	0.2±0.04 (0.19)	0.2±0.06 (0.21)
Kentucky	0.1±0.02 (0.1)	0.09±0.01 (0.09)	0.04±0.01 (0.04)	0.1±0.01 (0.1)	0.11±0.02 (0.11)	0.1±0.03 (0.1)	0.13±0.02 (0.13)	0.09±0.02 (0.09)	0.08±0.02 (0.08)	0.13±0.02 (0.13)
Louisiana	0.16±0.04 (0.15)	0.17±0.08 (0.15)	0.11±0.03 (0.1)	0.17±0.05 (0.16)	0.16±0.04 (0.15)	0.14±0.04 (0.13)	0.13±0.05 (0.12)	0.13±0.03 (0.13)	0.14±0.05 (0.12)	0.14±0.05 (0.13)
Mississippi	0.19±0.03 (0.18)	0.2±0.04 (0.2)	0.13±0.03 (0.12)	0.2±0.04 (0.19)	0.23±0.07 (0.22)	0.17±0.03 (0.17)	0.19±0.04 (0.18)	0.17±0.03 (0.16)	0.14±0.03 (0.14)	0.17±0.05 (0.16)
North Carolina	0.11±0.03 (0.11)	0.08±0.03 (0.07)	0.07±0.02 (0.06)	0.09±0.04 (0.09)	0.14±0.04 (0.13)	0.14±0.04 (0.13)	0.13±0.05 (0.12)	0.1±0.03 (0.09)	0.12±0.04 (0.11)	0.14±0.07 (0.13)
South Carolina	0.13±0.02 (0.13)	0.1±0.02 (0.1)	0.08±0.01 (0.08)	0.1±0.03 (0.1)	0.16±0.04 (0.16)	0.17±0.03 (0.17)	0.13±0.03 (0.13)	0.11±0.02 (0.11)	0.14±0.03 (0.14)	0.15±0.05 (0.15)
Tennessee	0.14±0.02 (0.14)	0.13±0.03 (0.13)	0.07±0.01 (0.07)	0.14±0.02 (0.14)	0.16±0.03 (0.16)	0.16±0.03 (0.16)	0.17±0.03 (0.17)	0.13±0.02 (0.12)	0.13±0.02 (0.12)	0.17±0.03 (0.17)
Virginia	0.09±0.02 (0.09)	0.07±0.02 (0.06)	0.04±0.01 (0.04)	0.08±0.02 (0.08)	0.1±0.03 (0.1)	0.1±0.04 (0.09)	0.12±0.04 (0.12)	0.08±0.02 (0.07)	0.09±0.02 (0.09)	0.12±0.04 (0.12)
West Virginia	0.07±0.02 (0.07)	0.06±0.02 (0.06)	0.04±0.01 (0.04)	0.07±0.01 (0.07)	0.08±0.03 (0.07)	0.06±0.02 (0.05)	0.1±0.02 (0.11)	0.06±0.01 (0.06)	0.06±0.02 (0.06)	0.08±0.02 (0.07)

Table S13: State-level moderate burning season (October-November) (OND) average prescribed burn smoke OC concentrations (μg/m^3).

	Average 2013-2021	2013	2014	2015	2016	2017	2018	2019	2020	2021
Alabama	0.61±0.11 (0.61)	0.44±0.08 (0.44)	0.5±0.16 (0.5)	0.4±0.09 (0.41)	0.46±0.08 (0.45)	0.58±0.13 (0.57)	0.6±0.16 (0.61)	0.82±0.17 (0.81)	0.81±0.17 (0.8)	0.65±0.18 (0.62)
Arkansas	0.3±0.05 (0.29)	0.17±0.04 (0.17)	0.17±0.05 (0.16)	0.14±0.03 (0.15)	0.37±0.1 (0.35)	0.34±0.05 (0.34)	0.16±0.05 (0.15)	0.32±0.11 (0.31)	0.51±0.11 (0.51)	0.24±0.06 (0.24)
Florida	0.3±0.15 (0.26)	0.17±0.11 (0.14)	0.42±0.14 (0.42)	0.13±0.11 (0.11)	0.27±0.14 (0.26)	0.31±0.2 (0.27)	0.19±0.12 (0.18)	0.46±0.21 (0.4)	0.26±0.23 (0.2)	0.29±0.18 (0.25)
Georgia	0.52±0.12 (0.52)	0.35±0.12 (0.33)	0.52±0.16 (0.52)	0.29±0.08 (0.29)	0.42±0.09 (0.41)	0.52±0.12 (0.54)	0.42±0.18 (0.39)	0.75±0.24 (0.73)	0.63±0.21 (0.63)	0.61±0.17 (0.61)
Kentucky	0.24±0.03 (0.24)	0.17±0.03 (0.17)	0.11±0.01 (0.11)	0.17±0.03 (0.17)	0.24±0.06 (0.23)	0.31±0.04 (0.31)	0.17±0.04 (0.17)	0.18±0.04 (0.18)	0.37±0.07 (0.39)	0.23±0.04 (0.23)
Louisiana	0.36±0.05 (0.35)	0.22±0.06 (0.21)	0.27±0.06 (0.27)	0.18±0.04 (0.17)	0.43±0.1 (0.43)	0.39±0.12 (0.35)	0.23±0.06 (0.22)	0.54±0.09 (0.52)	0.52±0.11 (0.5)	0.29±0.06 (0.28)
Mississippi	0.43±0.08 (0.43)	0.29±0.08 (0.28)	0.31±0.1 (0.31)	0.25±0.07 (0.23)	0.39±0.07 (0.38)	0.49±0.09 (0.47)	0.38±0.14 (0.37)	0.59±0.13 (0.59)	0.62±0.1 (0.6)	0.36±0.09 (0.35)
North Carolina	0.24±0.07 (0.24)	0.14±0.04 (0.13)	0.15±0.06 (0.14)	0.14±0.04 (0.13)	0.29±0.1 (0.28)	0.29±0.11 (0.29)	0.16±0.08 (0.16)	0.31±0.12 (0.31)	0.23±0.1 (0.23)	0.33±0.09 (0.32)
South Carolina	0.31±0.07 (0.31)	0.2±0.06 (0.2)	0.28±0.08 (0.27)	0.2±0.05 (0.21)	0.29±0.05 (0.28)	0.36±0.08 (0.38)	0.25±0.1 (0.24)	0.41±0.16 (0.35)	0.28±0.07 (0.27)	0.45±0.08 (0.43)
Tennessee	0.32±0.04 (0.32)	0.22±0.04 (0.22)	0.16±0.03 (0.16)	0.21±0.04 (0.22)	0.3±0.05 (0.29)	0.36±0.05 (0.36)	0.25±0.06 (0.25)	0.38±0.09 (0.37)	0.43±0.1 (0.46)	0.33±0.05 (0.33)
Virginia	0.18±0.04 (0.17)	0.12±0.03 (0.12)	0.12±0.04 (0.12)	0.1±0.02 (0.11)	0.21±0.06 (0.21)	0.22±0.08 (0.22)	0.09±0.03 (0.08)	0.17±0.07 (0.16)	0.2±0.04 (0.2)	0.2±0.05 (0.2)
West Virginia	0.16±0.02 (0.16)	0.11±0.02 (0.11)	0.1±0.02 (0.1)	0.09±0.02 (0.1)	0.16±0.04 (0.15)	0.24±0.04 (0.24)	0.08±0.02 (0.08)	0.13±0.04 (0.12)	0.22±0.03 (0.22)	0.15±0.04 (0.14)

Table S14: State-level yearly average prescribed burn smoke NO_3^- concentrations (μg/m^3).

	Average 2013-2021	2013	2014	2015	2016	2017	2018	2019	2020	2021
Alabama	0.014±0.006 (0.013)	0.019±0.008 (0.018)	0.022±0.009 (0.02)	0.014±0.004 (0.013)	0.009±0.003 (0.009)	0.01±0.006 (0.009)	0.012±0.005 (0.011)	0.012±0.006 (0.01)	0.01±0.004 (0.009)	0.015±0.008 (0.015)
Arkansas	0.008±0.002 (0.008)	0.015±0.005 (0.016)	0.01±0.003 (0.01)	0.007±0.002 (0.007)	0.006±0.002 (0.006)	0.005±0.002 (0.005)	0.009±0.003 (0.009)	0.009±0.004 (0.009)	0.007±0.003 (0.007)	0.006±0.002 (0.006)
Florida	0.006±0.002 (0.006)	0.007±0.004 (0.007)	0.009±0.004 (0.008)	0.006±0.002 (0.006)	0.004±0.002 (0.004)	0.005±0.002 (0.005)	0.008±0.003 (0.008)	0.006±0.002 (0.006)	0.003±0.001 (0.003)	0.005±0.003 (0.004)
Georgia	0.015±0.006 (0.014)	0.02±0.009 (0.018)	0.027±0.01 (0.027)	0.013±0.005 (0.013)	0.01±0.004 (0.01)	0.012±0.005 (0.011)	0.014±0.005 (0.013)	0.012±0.006 (0.01)	0.009±0.004 (0.007)	0.017±0.009 (0.014)
Kentucky	0.013±0.003 (0.014)	0.019±0.006 (0.02)	0.02±0.003 (0.02)	0.012±0.003 (0.012)	0.01±0.002 (0.009)	0.011±0.003 (0.011)	0.011±0.002 (0.011)	0.013±0.003 (0.012)	0.01±0.003 (0.011)	0.013±0.003 (0.014)
Louisiana	0.006±0.002 (0.006)	0.008±0.003 (0.007)	0.008±0.003 (0.008)	0.007±0.002 (0.007)	0.004±0.001 (0.004)	0.004±0.002 (0.004)	0.006±0.002 (0.006)	0.006±0.003 (0.006)	0.005±0.002 (0.004)	0.007±0.003 (0.005)
Mississippi	0.008±0.002 (0.008)	0.014±0.004 (0.014)	0.012±0.003 (0.011)	0.008±0.002 (0.008)	0.005±0.002 (0.005)	0.006±0.002 (0.005)	0.008±0.002 (0.007)	0.008±0.003 (0.008)	0.005±0.002 (0.005)	0.007±0.002 (0.007)
North Carolina	0.014±0.005 (0.014)	0.016±0.005 (0.017)	0.02±0.008 (0.02)	0.012±0.004 (0.013)	0.011±0.004 (0.012)	0.014±0.005 (0.014)	0.013±0.005 (0.012)	0.012±0.005 (0.012)	0.009±0.003 (0.009)	0.014±0.005 (0.015)
South Carolina	0.011±0.003 (0.011)	0.013±0.003 (0.013)	0.02±0.005 (0.019)	0.01±0.003 (0.01)	0.009±0.003 (0.008)	0.012±0.003 (0.011)	0.01±0.003 (0.01)	0.008±0.004 (0.008)	0.006±0.002 (0.006)	0.013±0.004 (0.012)
Tennessee	0.014±0.003 (0.014)	0.023±0.004 (0.023)	0.024±0.005 (0.023)	0.013±0.003 (0.012)	0.009±0.003 (0.01)	0.011±0.003 (0.011)	0.012±0.004 (0.011)	0.014±0.003 (0.014)	0.01±0.003 (0.01)	0.015±0.005 (0.014)
Virginia	0.009±0.003 (0.009)	0.01±0.003 (0.01)	0.014±0.004 (0.012)	0.009±0.002 (0.008)	0.009±0.002 (0.008)	0.009±0.003 (0.008)	0.009±0.003 (0.008)	0.009±0.004 (0.008)	0.007±0.001 (0.007)	0.01±0.003 (0.008)
West Virginia	0.008±0.002 (0.008)	0.01±0.002 (0.01)	0.013±0.003 (0.012)	0.007±0.002 (0.007)	0.007±0.002 (0.007)	0.008±0.002 (0.007)	0.008±0.002 (0.008)	0.009±0.003 (0.009)	0.005±0.001 (0.005)	0.008±0.002 (0.007)

Table S15: State-level extensive burning season (January-April) (JFMA) average prescribed burn smoke NO_3^- concentrations (µg/m^3).

	Average 2013-2021	2013	2014	2015	2016	2017	2018	2019	2020	2021
Alabama	0.02±0.008 (0.019)	0.03±0.012 (0.028)	0.038±0.02 (0.034)	0.022±0.006 (0.021)	0.016±0.006 (0.015)	0.012±0.007 (0.01)	0.018±0.007 (0.017)	0.021±0.011 (0.018)	0.01±0.004 (0.009)	0.017±0.009 (0.016)
Arkansas	0.013±0.005 (0.013)	0.027±0.01 (0.028)	0.017±0.007 (0.016)	0.011±0.004 (0.01)	0.008±0.004 (0.008)	0.006±0.003 (0.006)	0.016±0.006 (0.016)	0.016±0.009 (0.015)	0.005±0.003 (0.005)	0.008±0.003 (0.008)
Florida	0.01±0.004 (0.01)	0.016±0.009 (0.015)	0.011±0.006 (0.01)	0.013±0.005 (0.013)	0.007±0.003 (0.007)	0.007±0.003 (0.007)	0.015±0.007 (0.015)	0.009±0.004 (0.009)	0.005±0.002 (0.004)	0.007±0.003 (0.006)
Georgia	0.026±0.009 (0.026)	0.034±0.012 (0.035)	0.052±0.022 (0.05)	0.025±0.008 (0.025)	0.02±0.007 (0.021)	0.019±0.008 (0.018)	0.026±0.006 (0.026)	0.025±0.014 (0.021)	0.01±0.004 (0.01)	0.024±0.012 (0.02)
Kentucky	0.021±0.004 (0.022)	0.03±0.012 (0.028)	0.042±0.008 (0.042)	0.014±0.004 (0.014)	0.016±0.004 (0.015)	0.013±0.004 (0.013)	0.018±0.004 (0.018)	0.027±0.009 (0.026)	0.01±0.002 (0.01)	0.022±0.006 (0.022)
Louisiana	0.007±0.002 (0.007)	0.009±0.003 (0.009)	0.011±0.004 (0.011)	0.01±0.004 (0.009)	0.005±0.002 (0.004)	0.003±0.002 (0.002)	0.01±0.003 (0.009)	0.008±0.004 (0.007)	0.003±0.002 (0.002)	0.007±0.003 (0.006)
Mississippi	0.011±0.004 (0.011)	0.019±0.007 (0.018)	0.019±0.009 (0.016)	0.013±0.003 (0.012)	0.007±0.003 (0.007)	0.006±0.003 (0.005)	0.012±0.003 (0.012)	0.013±0.005 (0.011)	0.004±0.002 (0.004)	0.008±0.003 (0.008)
North Carolina	0.021±0.007 (0.022)	0.027±0.009 (0.028)	0.039±0.015 (0.037)	0.019±0.006 (0.019)	0.017±0.006 (0.016)	0.019±0.006 (0.02)	0.018±0.008 (0.019)	0.021±0.01 (0.02)	0.011±0.004 (0.012)	0.02±0.007 (0.021)
South Carolina	0.02±0.004 (0.02)	0.026±0.005 (0.025)	0.04±0.01 (0.038)	0.018±0.004 (0.017)	0.017±0.005 (0.017)	0.02±0.004 (0.019)	0.019±0.003 (0.018)	0.016±0.006 (0.014)	0.008±0.002 (0.008)	0.019±0.005 (0.018)
Tennessee	0.023±0.005 (0.023)	0.039±0.009 (0.039)	0.049±0.01 (0.049)	0.016±0.004 (0.015)	0.015±0.005 (0.016)	0.013±0.004 (0.013)	0.019±0.006 (0.018)	0.025±0.007 (0.025)	0.01±0.003 (0.01)	0.02±0.007 (0.018)
Virginia	0.015±0.004 (0.014)	0.014±0.005 (0.013)	0.027±0.009 (0.023)	0.013±0.003 (0.012)	0.014±0.004 (0.013)	0.012±0.003 (0.011)	0.014±0.006 (0.012)	0.017±0.008 (0.015)	0.009±0.001 (0.009)	0.014±0.004 (0.013)
West Virginia	0.014±0.003 (0.013)	0.013±0.004 (0.013)	0.026±0.006 (0.025)	0.012±0.004 (0.011)	0.012±0.003 (0.012)	0.01±0.002 (0.009)	0.014±0.003 (0.013)	0.019±0.005 (0.018)	0.006±0.001 (0.006)	0.013±0.002 (0.013)

Table S16: State-level low burning season (May-September) (MJJAS) average prescribed burn smoke NO_3^- concentrations (µg/m^3).

	Average 2013-2021	2013	2014	2015	2016	2017	2018	2019	2020	2021
Alabama	0.006±0.003 (0.005)	0.007±0.004 (0.006)	0.005±0.003 (0.004)	0.006±0.003 (0.005)	0.004±0.002 (0.004)	0.005±0.004 (0.004)	0.008±0.005 (0.006)	0.005±0.004 (0.004)	0.003±0.002 (0.002)	0.01±0.007 (0.008)
Arkansas	0.004±0.001 (0.004)	0.006±0.002 (0.005)	0.004±0.001 (0.004)	0.004±0.002 (0.004)	0.004±0.002 (0.003)	0.003±0.002 (0.003)	0.005±0.002 (0.004)	0.004±0.002 (0.003)	0.002±0.001 (0.002)	0.003±0.001 (0.003)
Florida	0.003±0.001 (0.003)	0.002±0.001 (0.002)	0.004±0.002 (0.004)	0.003±0.001 (0.003)	0.003±0.002 (0.003)	0.004±0.002 (0.003)	0.004±0.002 (0.004)	0.004±0.003 (0.004)	0.002±0.001 (0.002)	0.002±0.001 (0.002)
Georgia	0.005±0.003 (0.004)	0.006±0.004 (0.004)	0.005±0.003 (0.004)	0.004±0.003 (0.003)	0.004±0.002 (0.003)	0.005±0.002 (0.004)	0.006±0.004 (0.005)	0.004±0.002 (0.003)	0.003±0.002 (0.002)	0.008±0.005 (0.006)
Kentucky	0.005±0.002 (0.005)	0.007±0.003 (0.008)	0.004±0.002 (0.004)	0.006±0.002 (0.006)	0.005±0.002 (0.005)	0.005±0.002 (0.005)	0.007±0.002 (0.007)	0.005±0.001 (0.005)	0.003±0.001 (0.003)	0.007±0.002 (0.006)
Louisiana	0.004±0.001 (0.003)	0.005±0.002 (0.004)	0.004±0.003 (0.003)	0.005±0.002 (0.004)	0.003±0.001 (0.003)	0.003±0.001 (0.003)	0.004±0.002 (0.004)	0.004±0.002 (0.003)	0.002±0.001 (0.002)	0.004±0.003 (0.003)
Mississippi	0.004±0.001 (0.004)	0.006±0.002 (0.006)	0.004±0.002 (0.004)	0.004±0.002 (0.004)	0.003±0.001 (0.003)	0.003±0.001 (0.003)	0.005±0.002 (0.005)	0.004±0.001 (0.003)	0.002±0.001 (0.001)	0.004±0.002 (0.003)
North Carolina	0.006±0.003 (0.006)	0.005±0.002 (0.005)	0.007±0.004 (0.006)	0.006±0.003 (0.005)	0.007±0.004 (0.006)	0.006±0.004 (0.006)	0.01±0.005 (0.009)	0.006±0.004 (0.006)	0.004±0.002 (0.003)	0.006±0.004 (0.006)
South Carolina	0.004±0.001 (0.003)	0.003±0.001 (0.003)	0.003±0.002 (0.003)	0.003±0.001 (0.002)	0.003±0.001 (0.003)	0.004±0.001 (0.004)	0.005±0.002 (0.005)	0.004±0.001 (0.003)	0.002±0.001 (0.002)	0.005±0.002 (0.004)
Tennessee	0.006±0.002 (0.005)	0.008±0.002 (0.008)	0.005±0.001 (0.005)	0.006±0.002 (0.006)	0.004±0.002 (0.004)	0.005±0.002 (0.005)	0.008±0.003 (0.007)	0.005±0.002 (0.005)	0.003±0.001 (0.003)	0.008±0.003 (0.007)
Virginia	0.004±0.001 (0.003)	0.004±0.001 (0.003)	0.003±0.001 (0.003)	0.004±0.002 (0.003)	0.004±0.002 (0.004)	0.003±0.002 (0.003)	0.007±0.003 (0.007)	0.004±0.002 (0.004)	0.002±0.001 (0.002)	0.004±0.003 (0.003)
West Virginia	0.003±0.001 (0.003)	0.004±0.001 (0.004)	0.003±0.002 (0.003)	0.003±0.001 (0.003)	0.003±0.001 (0.003)	0.002±0.001 (0.002)	0.006±0.002 (0.005)	0.003±0.001 (0.003)	0.002±0.001 (0.002)	0.003±0.002 (0.003)

Table S17: State-level moderate burning season (October-November) (OND) average prescribed burn smoke NO_3^- concentrations (μg/m^3).

	Average 2013-2021	2013	2014	2015	2016	2017	2018	2019	2020	2021
Alabama	0.018±0.00 7 (0.017)	0.025±0.01 1 (0.023)	0.029±0.00 9 (0.028)	0.017±0.00 8 (0.016)	0.007±0.00 3 (0.006)	0.016±0.00 7 (0.015)	0.011±0.00 4 (0.01)	0.011±0.00 5 (0.01)	0.02±0.01 (0.017)	0.023±0.01 (0.021)
Arkansas	0.01±0.003 (0.01)	0.017±0.00 5 (0.016)	0.014±0.00 5 (0.013)	0.009±0.00 4 (0.008)	0.007±0.00 2 (0.007)	0.007±0.00 2 (0.007)	0.006±0.00 3 (0.005)	0.009±0.00 3 (0.008)	0.017±0.00 7 (0.016)	0.009±0.00 2 (0.008)
Florida	0.005±0.00 3 (0.004)	0.004±0.00 3 (0.003)	0.014±0.00 8 (0.011)	0.003±0.00 2 (0.003)	0.003±0.00 1 (0.003)	0.005±0.00 3 (0.004)	0.003±0.00 1 (0.002)	0.004±0.00 1 (0.003)	0.004±0.00 3 (0.003)	0.006±0.00 5 (0.005)
Georgia	0.017±0.00 8 (0.015)	0.024±0.01 9 (0.018)	0.032±0.01 1 (0.033)	0.014±0.00 9 (0.01)	0.008±0.00 4 (0.007)	0.015±0.00 8 (0.013)	0.01±0.005 (0.01)	0.01±0.005 (0.008)	0.016±0.00 9 (0.013)	0.024±0.01 3 (0.02)
Kentucky	0.017±0.00 4 (0.018)	0.028±0.00 8 (0.03)	0.018±0.00 4 (0.019)	0.019±0.00 6 (0.02)	0.01±0.003 (0.01)	0.02±0.004 (0.02)	0.009±0.00 3 (0.009)	0.009±0.00 2 (0.009)	0.023±0.00 8 (0.024)	0.015±0.00 4 (0.015)
Louisiana	0.009±0.00 3 (0.008)	0.011±0.00 5 (0.01)	0.012±0.00 4 (0.011)	0.006±0.00 2 (0.006)	0.005±0.00 2 (0.005)	0.008±0.00 4 (0.007)	0.005±0.00 3 (0.004)	0.009±0.00 4 (0.008)	0.011±0.00 5 (0.01)	0.011±0.00 7 (0.009)
Mississipp i	0.011±0.00 3 (0.011)	0.019±0.00 7 (0.018)	0.017±0.00 4 (0.016)	0.01±0.004 (0.009)	0.006±0.00 2 (0.006)	0.01±0.003 (0.01)	0.007±0.00 2 (0.007)	0.009±0.00 3 (0.009)	0.013±0.00 4 (0.012)	0.01±0.002 (0.01)
North Carolina	0.016±0.00 7 (0.017)	0.02±0.01 (0.02)	0.02±0.011 (0.019)	0.015±0.00 6 (0.015)	0.013±0.00 5 (0.013)	0.018±0.00 8 (0.018)	0.009±0.00 5 (0.009)	0.011±0.00 6 (0.011)	0.013±0.00 8 (0.013)	0.018±0.00 8 (0.02)
South Carolina	0.013±0.00 4 (0.013)	0.014±0.00 7 (0.013)	0.021±0.00 7 (0.02)	0.014±0.00 5 (0.014)	0.007±0.00 2 (0.007)	0.014±0.00 5 (0.014)	0.008±0.00 3 (0.008)	0.007±0.00 4 (0.006)	0.01±0.003 (0.01)	0.017±0.00 6 (0.017)
Tennessee	0.019±0.00 5 (0.018)	0.03±0.007 (0.03)	0.023±0.00 6 (0.022)	0.02±0.006 (0.021)	0.011±0.00 3 (0.011)	0.019±0.00 6 (0.018)	0.01±0.004 (0.009)	0.013±0.00 4 (0.013)	0.023±0.00 7 (0.022)	0.021±0.00 8 (0.02)
Virginia	0.012±0.00 3 (0.012)	0.017±0.00 4 (0.017)	0.015±0.00 6 (0.014)	0.012±0.00 3 (0.012)	0.011±0.00 4 (0.01)	0.013±0.00 5 (0.013)	0.005±0.00 2 (0.005)	0.008±0.00 4 (0.007)	0.013±0.00 4 (0.014)	0.013±0.00 5 (0.011)
West Virginia	0.01±0.002 (0.01)	0.018±0.00 4 (0.018)	0.013±0.00 4 (0.012)	0.009±0.00 2 (0.009)	0.009±0.00 3 (0.008)	0.014±0.00 4 (0.013)	0.005±0.00 1 (0.004)	0.007±0.00 3 (0.006)	0.01±0.003 (0.01)	0.009±0.00 3 (0.008)

Table S18: State-level yearly average prescribed burn smoke SO_4^{2-} concentrations (µg/m^3).

	Average 2013-2021	2013	2014	2015	2016	2017	2018	2019	2020	2021
Alabama	0.031±0.004 (0.031)	0.04±0.004 (0.041)	0.04±0.005 (0.041)	0.032±0.005 (0.033)	0.028±0.003 (0.028)	0.033±0.005 (0.032)	0.023±0.002 (0.023)	0.028±0.003 (0.028)	0.022±0.003 (0.022)	0.03±0.006 (0.029)
Arkansas	0.019±0.002 (0.019)	0.028±0.003 (0.029)	0.021±0.003 (0.021)	0.022±0.003 (0.022)	0.019±0.003 (0.019)	0.019±0.001 (0.019)	0.016±0.002 (0.016)	0.018±0.002 (0.018)	0.015±0.002 (0.015)	0.016±0.002 (0.016)
Florida	0.025±0.006 (0.025)	0.029±0.008 (0.029)	0.034±0.007 (0.036)	0.031±0.006 (0.031)	0.021±0.006 (0.022)	0.025±0.007 (0.026)	0.018±0.004 (0.019)	0.023±0.005 (0.022)	0.019±0.006 (0.02)	0.021±0.007 (0.022)
Georgia	0.029±0.004 (0.029)	0.033±0.005 (0.033)	0.039±0.006 (0.039)	0.028±0.004 (0.027)	0.028±0.003 (0.028)	0.033±0.005 (0.033)	0.021±0.002 (0.021)	0.026±0.003 (0.026)	0.022±0.005 (0.021)	0.03±0.004 (0.03)
Kentucky	0.019±0.001 (0.019)	0.027±0.002 (0.027)	0.021±0.002 (0.021)	0.021±0.002 (0.021)	0.018±0.001 (0.018)	0.017±0.001 (0.018)	0.016±0.001 (0.016)	0.017±0.001 (0.017)	0.012±0.001 (0.012)	0.018±0.002 (0.018)
Louisiana	0.023±0.001 (0.023)	0.031±0.004 (0.031)	0.029±0.003 (0.029)	0.028±0.003 (0.028)	0.022±0.002 (0.022)	0.021±0.002 (0.021)	0.018±0.001 (0.018)	0.022±0.001 (0.022)	0.018±0.002 (0.017)	0.017±0.001 (0.017)
Mississippi	0.024±0.002 (0.024)	0.036±0.003 (0.035)	0.032±0.005 (0.031)	0.029±0.003 (0.029)	0.022±0.002 (0.022)	0.025±0.003 (0.024)	0.018±0.002 (0.018)	0.023±0.002 (0.023)	0.017±0.002 (0.017)	0.019±0.003 (0.019)
North Carolina	0.018±0.003 (0.019)	0.02±0.003 (0.02)	0.023±0.004 (0.023)	0.018±0.003 (0.019)	0.02±0.003 (0.021)	0.018±0.003 (0.018)	0.014±0.002 (0.014)	0.018±0.003 (0.018)	0.014±0.002 (0.014)	0.018±0.003 (0.019)
South Carolina	0.022±0.002 (0.022)	0.025±0.003 (0.025)	0.031±0.003 (0.031)	0.022±0.002 (0.022)	0.023±0.002 (0.024)	0.025±0.003 (0.025)	0.016±0.001 (0.016)	0.021±0.002 (0.021)	0.016±0.001 (0.016)	0.023±0.003 (0.023)
Tennessee	0.022±0.002 (0.021)	0.03±0.003 (0.03)	0.026±0.003 (0.026)	0.024±0.001 (0.024)	0.021±0.002 (0.021)	0.021±0.002 (0.021)	0.018±0.002 (0.018)	0.021±0.002 (0.021)	0.014±0.001 (0.015)	0.021±0.003 (0.021)
Virginia	0.015±0.002 (0.015)	0.018±0.003 (0.017)	0.017±0.003 (0.016)	0.016±0.003 (0.016)	0.017±0.003 (0.017)	0.014±0.002 (0.014)	0.013±0.002 (0.012)	0.014±0.003 (0.013)	0.012±0.001 (0.012)	0.015±0.003 (0.014)
West Virginia	0.015±0.002 (0.016)	0.022±0.003 (0.022)	0.017±0.002 (0.018)	0.018±0.002 (0.018)	0.015±0.002 (0.015)	0.014±0.002 (0.013)	0.014±0.002 (0.014)	0.014±0.002 (0.014)	0.01±0.001 (0.01)	0.014±0.002 (0.014)

Table S19: State-level extensive burning season (January-April) (JFMA) average prescribed burn smoke SO_4^{2-} concentrations (μg/m³).

	Average 2013-2021	2013	2014	2015	2016	2017	2018	2019	2020	2021
Alabama	0.03±0.005 (0.029)	0.038±0.006 (0.039)	0.036±0.006 (0.036)	0.024±0.008 (0.025)	0.026±0.004 (0.025)	0.042±0.009 (0.041)	0.025±0.005 (0.024)	0.026±0.005 (0.026)	0.019±0.004 (0.018)	0.033±0.01 (0.029)
Arkansas	0.013±0.001 (0.013)	0.017±0.002 (0.016)	0.015±0.003 (0.016)	0.009±0.003 (0.009)	0.01±0.001 (0.01)	0.017±0.003 (0.017)	0.015±0.002 (0.015)	0.011±0.001 (0.011)	0.007±0.001 (0.007)	0.014±0.002 (0.014)
Florida	0.033±0.007 (0.034)	0.044±0.009 (0.044)	0.038±0.01 (0.04)	0.051±0.012 (0.051)	0.022±0.005 (0.022)	0.034±0.007 (0.033)	0.029±0.006 (0.03)	0.029±0.009 (0.028)	0.025±0.007 (0.025)	0.029±0.009 (0.028)
Georgia	0.036±0.006 (0.036)	0.038±0.008 (0.038)	0.048±0.007 (0.047)	0.029±0.008 (0.029)	0.033±0.005 (0.033)	0.047±0.009 (0.048)	0.029±0.004 (0.029)	0.031±0.005 (0.032)	0.026±0.008 (0.024)	0.042±0.006 (0.042)
Kentucky	0.017±0.002 (0.017)	0.025±0.003 (0.025)	0.022±0.003 (0.022)	0.01±0.002 (0.01)	0.019±0.003 (0.02)	0.018±0.002 (0.018)	0.014±0.002 (0.015)	0.017±0.001 (0.017)	0.009±0.001 (0.009)	0.019±0.004 (0.019)
Louisiana	0.018±0.002 (0.018)	0.022±0.004 (0.022)	0.025±0.006 (0.025)	0.02±0.005 (0.019)	0.013±0.002 (0.012)	0.018±0.004 (0.018)	0.019±0.003 (0.018)	0.015±0.003 (0.015)	0.012±0.003 (0.012)	0.016±0.002 (0.016)
Mississippi	0.02±0.003 (0.019)	0.026±0.005 (0.025)	0.024±0.004 (0.025)	0.018±0.005 (0.016)	0.015±0.003 (0.014)	0.029±0.005 (0.028)	0.017±0.002 (0.017)	0.017±0.003 (0.017)	0.012±0.003 (0.011)	0.018±0.003 (0.018)
North Carolina	0.021±0.003 (0.021)	0.021±0.004 (0.02)	0.028±0.005 (0.028)	0.016±0.003 (0.016)	0.022±0.002 (0.023)	0.024±0.005 (0.024)	0.015±0.003 (0.015)	0.019±0.003 (0.019)	0.017±0.004 (0.017)	0.025±0.004 (0.025)
South Carolina	0.028±0.003 (0.027)	0.029±0.005 (0.027)	0.039±0.004 (0.04)	0.021±0.003 (0.02)	0.027±0.003 (0.027)	0.035±0.006 (0.034)	0.02±0.002 (0.02)	0.024±0.003 (0.024)	0.019±0.003 (0.02)	0.034±0.004 (0.034)
Tennessee	0.02±0.003 (0.02)	0.025±0.003 (0.026)	0.028±0.004 (0.028)	0.014±0.002 (0.014)	0.02±0.005 (0.022)	0.024±0.003 (0.023)	0.017±0.004 (0.017)	0.018±0.002 (0.018)	0.011±0.001 (0.011)	0.02±0.005 (0.02)
Virginia	0.016±0.002 (0.015)	0.017±0.003 (0.016)	0.019±0.004 (0.018)	0.011±0.002 (0.011)	0.019±0.002 (0.019)	0.019±0.002 (0.019)	0.014±0.002 (0.014)	0.014±0.003 (0.013)	0.01±0.002 (0.01)	0.018±0.003 (0.018)
West Virginia	0.015±0.002 (0.015)	0.019±0.003 (0.019)	0.018±0.003 (0.018)	0.011±0.002 (0.01)	0.017±0.002 (0.017)	0.015±0.001 (0.015)	0.015±0.002 (0.015)	0.014±0.002 (0.014)	0.007±0.002 (0.007)	0.016±0.002 (0.016)

Table S20: State-level low burning season (May-September) (MJJAS) average prescribed burn smoke SO_4^{2-} concentrations (µg/m^3).

	Average 2013-2021	2013	2014	2015	2016	2017	2018	2019	2020	2021
Alabama	0.03±0.003 (0.03)	0.042±0.006 (0.042)	0.036±0.006 (0.036)	0.036±0.005 (0.036)	0.031±0.004 (0.031)	0.024±0.003 (0.025)	0.024±0.002 (0.024)	0.033±0.003 (0.033)	0.016±0.002 (0.016)	0.025±0.005 (0.024)
Arkansas	0.024±0.003 (0.024)	0.038±0.006 (0.038)	0.025±0.004 (0.024)	0.033±0.004 (0.034)	0.023±0.004 (0.023)	0.018±0.003 (0.017)	0.02±0.003 (0.02)	0.024±0.003 (0.024)	0.014±0.002 (0.014)	0.016±0.002 (0.016)
Florida	0.019±0.006 (0.02)	0.02±0.012 (0.021)	0.026±0.007 (0.027)	0.022±0.008 (0.022)	0.02±0.007 (0.022)	0.019±0.007 (0.021)	0.015±0.006 (0.014)	0.021±0.006 (0.02)	0.014±0.005 (0.016)	0.012±0.006 (0.011)
Georgia	0.024±0.002 (0.024)	0.031±0.004 (0.031)	0.029±0.004 (0.029)	0.026±0.004 (0.027)	0.027±0.003 (0.028)	0.022±0.003 (0.022)	0.019±0.003 (0.019)	0.027±0.003 (0.026)	0.017±0.002 (0.017)	0.021±0.004 (0.021)
Kentucky	0.019±0.002 (0.019)	0.028±0.003 (0.028)	0.019±0.002 (0.019)	0.027±0.003 (0.027)	0.018±0.002 (0.018)	0.015±0.002 (0.015)	0.018±0.002 (0.018)	0.021±0.002 (0.022)	0.01±0.001 (0.01)	0.016±0.002 (0.017)
Louisiana	0.026±0.003 (0.025)	0.04±0.009 (0.041)	0.032±0.004 (0.032)	0.037±0.004 (0.036)	0.026±0.003 (0.026)	0.019±0.002 (0.019)	0.02±0.002 (0.02)	0.027±0.003 (0.027)	0.014±0.002 (0.014)	0.014±0.002 (0.013)
Mississippi	0.027±0.002 (0.027)	0.044±0.005 (0.045)	0.034±0.005 (0.034)	0.037±0.003 (0.037)	0.027±0.004 (0.027)	0.02±0.003 (0.019)	0.021±0.002 (0.021)	0.028±0.002 (0.028)	0.013±0.001 (0.013)	0.016±0.003 (0.016)
North Carolina	0.017±0.003 (0.018)	0.018±0.004 (0.018)	0.02±0.004 (0.02)	0.02±0.005 (0.019)	0.021±0.004 (0.021)	0.014±0.003 (0.014)	0.016±0.002 (0.016)	0.02±0.004 (0.021)	0.012±0.002 (0.012)	0.013±0.004 (0.013)
South Carolina	0.02±0.002 (0.02)	0.022±0.002 (0.022)	0.025±0.003 (0.025)	0.022±0.003 (0.021)	0.023±0.003 (0.024)	0.018±0.002 (0.019)	0.015±0.001 (0.015)	0.022±0.002 (0.022)	0.013±0.002 (0.014)	0.015±0.003 (0.015)
Tennessee	0.022±0.001 (0.023)	0.032±0.004 (0.032)	0.022±0.003 (0.022)	0.03±0.003 (0.03)	0.022±0.002 (0.022)	0.017±0.002 (0.017)	0.02±0.002 (0.02)	0.026±0.003 (0.026)	0.013±0.001 (0.012)	0.019±0.002 (0.019)
Virginia	0.015±0.003 (0.014)	0.016±0.004 (0.015)	0.015±0.003 (0.015)	0.021±0.004 (0.022)	0.016±0.003 (0.015)	0.011±0.002 (0.011)	0.016±0.003 (0.015)	0.017±0.003 (0.017)	0.01±0.002 (0.01)	0.013±0.003 (0.012)
West Virginia	0.016±0.002 (0.016)	0.022±0.004 (0.023)	0.017±0.003 (0.018)	0.023±0.002 (0.023)	0.015±0.003 (0.015)	0.01±0.002 (0.01)	0.016±0.002 (0.016)	0.017±0.002 (0.018)	0.009±0.001 (0.009)	0.012±0.002 (0.012)

Table S21: State-level moderate burning season (October-November) (OND) average prescribed burn smoke SO_4^{2-} concentrations (µg/m^3).

	Average 2013-2021	2013	2014	2015	2016	2017	2018	2019	2020	2021
Alabama	0.033±0.004 (0.034)	0.039±0.005 (0.039)	0.054±0.009 (0.054)	0.036±0.007 (0.036)	0.025±0.004 (0.024)	0.034±0.006 (0.033)	0.019±0.003 (0.019)	0.023±0.003 (0.023)	0.035±0.007 (0.035)	0.037±0.005 (0.038)
Arkansas	0.02±0.003 (0.021)	0.026±0.004 (0.027)	0.024±0.003 (0.024)	0.017±0.004 (0.016)	0.024±0.005 (0.022)	0.022±0.003 (0.022)	0.01±0.003 (0.011)	0.016±0.003 (0.015)	0.027±0.004 (0.026)	0.018±0.004 (0.019)
Florida	0.021±0.006 (0.02)	0.02±0.005 (0.02)	0.042±0.007 (0.041)	0.016±0.007 (0.014)	0.02±0.006 (0.019)	0.024±0.009 (0.023)	0.009±0.003 (0.009)	0.015±0.005 (0.014)	0.017±0.01 (0.015)	0.026±0.01 (0.027)
Georgia	0.027±0.004 (0.027)	0.03±0.008 (0.03)	0.046±0.01 (0.044)	0.028±0.005 (0.028)	0.023±0.002 (0.023)	0.03±0.006 (0.031)	0.014±0.004 (0.014)	0.019±0.003 (0.02)	0.025±0.006 (0.024)	0.031±0.004 (0.031)
Kentucky	0.02±0.002 (0.02)	0.029±0.004 (0.03)	0.022±0.003 (0.022)	0.024±0.002 (0.024)	0.017±0.002 (0.017)	0.021±0.002 (0.02)	0.014±0.002 (0.014)	0.01±0.001 (0.01)	0.021±0.003 (0.021)	0.019±0.002 (0.019)
Louisiana	0.025±0.002 (0.025)	0.027±0.005 (0.026)	0.029±0.005 (0.028)	0.021±0.004 (0.021)	0.029±0.005 (0.029)	0.029±0.004 (0.029)	0.011±0.002 (0.011)	0.023±0.003 (0.022)	0.029±0.004 (0.028)	0.024±0.004 (0.024)
Mississippi	0.026±0.003 (0.026)	0.032±0.006 (0.03)	0.036±0.008 (0.036)	0.028±0.006 (0.027)	0.022±0.003 (0.022)	0.027±0.003 (0.027)	0.015±0.003 (0.014)	0.02±0.003 (0.021)	0.03±0.003 (0.03)	0.027±0.004 (0.027)
North Carolina	0.017±0.003 (0.017)	0.023±0.004 (0.023)	0.019±0.006 (0.019)	0.019±0.003 (0.018)	0.019±0.003 (0.019)	0.016±0.004 (0.017)	0.008±0.003 (0.008)	0.013±0.002 (0.013)	0.015±0.004 (0.014)	0.019±0.004 (0.02)
South Carolina	0.021±0.002 (0.021)	0.025±0.004 (0.025)	0.031±0.005 (0.03)	0.023±0.003 (0.022)	0.019±0.002 (0.019)	0.023±0.004 (0.022)	0.01±0.003 (0.01)	0.014±0.002 (0.013)	0.015±0.002 (0.015)	0.025±0.002 (0.025)
Tennessee	0.023±0.002 (0.023)	0.03±0.005 (0.03)	0.029±0.005 (0.028)	0.025±0.003 (0.025)	0.019±0.001 (0.019)	0.024±0.002 (0.023)	0.015±0.002 (0.015)	0.015±0.002 (0.015)	0.023±0.005 (0.024)	0.025±0.002 (0.024)
Virginia	0.015±0.002 (0.015)	0.023±0.003 (0.023)	0.017±0.004 (0.016)	0.015±0.002 (0.015)	0.017±0.003 (0.016)	0.013±0.005 (0.012)	0.006±0.002 (0.005)	0.009±0.002 (0.008)	0.017±0.003 (0.017)	0.015±0.003 (0.015)
West Virginia	0.015±0.001 (0.016)	0.025±0.002 (0.024)	0.017±0.003 (0.017)	0.017±0.003 (0.017)	0.015±0.002 (0.015)	0.017±0.002 (0.017)	0.008±0.001 (0.008)	0.009±0.001 (0.009)	0.017±0.001 (0.017)	0.015±0.002 (0.015)
